# Cohesin Core Complex Gene Dosage Contributes to Germinal Center Derived Lymphoma Phenotypes and Outcomes

**DOI:** 10.3389/fimmu.2021.688493

**Published:** 2021-09-21

**Authors:** Martin A. Rivas, Ceyda Durmaz, Andreas Kloetgen, Cristopher R. Chin, Zhengming Chen, Bhavneet Bhinder, Amnon Koren, Aaron D. Viny, Christopher D. Scharer, Jeremy M. Boss, Olivier Elemento, Christopher E. Mason, Ari M. Melnick

**Affiliations:** ^1^Division of Hematology and Medical Oncology, Department of Medicine, Weill Cornell Medicine, New York, NY, United States; ^2^Graduate Program on Physiology, Biophysics & Systems Biology, Weill Cornell Medicine, New York, NY, United States; ^3^Department of Computational Biology of Infection Research, Helmholtz Centre for Infection Research, Braunschweig, Germany; ^4^Department of Physiology and Biophysics, Weill Cornell Medicine, New York, NY, United States; ^5^The HRH Prince Alwaleed Bin Talal Bin Abdulaziz Al-Saud Institute for Computational Biomedicine, Weill Cornell Medicine, New York, NY, United States; ^6^Division of Biostatistics and Epidemiology, Department of Population Health Sciences, Weill Cornell Medical College, New York, NY, United States; ^7^Caryl and Israel Englander Institute for Precision Medicine, Weill Cornell Medicine, New York, NY, United States; ^8^Department of Molecular Biology and Genetics, Cornell University, Ithaca, NY, United States; ^9^Division of Hematology/Oncology, Department of Medicine, Columbia University Irving Medical Center, New York, NY, United States; ^10^Columbia Stem Cell Initiative, Department of Genetics & Development, Columbia University, New York, NY, United States; ^11^Department of Microbiology and Immunology, School of Medicine, Emory University, Atlanta, GA, United States; ^12^The WorldQuant Initiative for Quantitative Prediction, Weill Cornell Medicine, New York, NY, United States; ^13^The Feil Family Brain and Mind Research Institute, Weill Cornell Medicine, New York, NY, United States

**Keywords:** cohesin, lymphoma, B-cell, chromosomal architecture, Hi-C, Tet2 gene, GCB-subtype DLBCL

## Abstract

The cohesin complex plays critical roles in genomic stability and gene expression through effects on 3D architecture. Cohesin core subunit genes are mutated across a wide cross-section of cancers, but not in germinal center (GC) derived lymphomas. In spite of this, haploinsufficiency of cohesin ATPase subunit *Smc3* was shown to contribute to malignant transformation of GC B-cells in mice. Herein we explored potential mechanisms and clinical relevance of *Smc3* deficiency in GC lymphomagenesis. Transcriptional profiling of *Smc3* haploinsufficient murine lymphomas revealed downregulation of genes repressed by loss of epigenetic tumor suppressors *Tet2* and *Kmt2d*. Profiling 3D chromosomal interactions in lymphomas revealed impaired enhancer-promoter interactions affecting genes like *Tet2*, which was aberrantly downregulated in *Smc3* deficient lymphomas. *Tet2* plays important roles in B-cell exit from the GC reaction, and single cell RNA-seq profiles and phenotypic trajectory analysis in *Smc3* mutant mice revealed a specific defect in commitment to the final steps of plasma cell differentiation. Although *Smc3* deficiency resulted in structural abnormalities in GC B-cells, there was no increase of somatic mutations or structural variants in *Smc3* haploinsufficient lymphomas, suggesting that cohesin deficiency largely induces lymphomas through disruption of enhancer-promoter interactions of terminal differentiation and tumor suppressor genes. Strikingly, the presence of the *Smc3* haploinsufficient GC B-cell transcriptional signature in human patients with GC-derived diffuse large B-cell lymphoma (DLBCL) was linked to inferior clinical outcome and low expression of cohesin core subunits. Reciprocally, reduced expression of cohesin subunits was an independent risk factor for worse survival int DLBCL patient cohorts. Collectively, the data suggest that *Smc3* functions as a bona fide tumor suppressor for lymphomas through non-genetic mechanisms, and drives disease by disrupting the commitment of GC B-cells to the plasma cell fate.

## Introduction

Cohesin proteins form a ring-shaped complex that plays a key role in 3D architectural organization of the genome, and is composed of Smc3, Smc1a, Stag1 or Stag2 and Rad21 subunits. Cohesin functions include maintaining sister chromatids cohesion until the end of mitosis, as well as maintaining chromatids aligned when DNA-damage occurs (1). Acting in concert with CCCTC-binding factor (CTCF), the cohesin complex forms chromatin regulatory structures, such topologically associated domains, and long distance interactions between gene regulatory elements such as enhancers with gene promoters, thus contributing transcriptional regulatory states and cell phenotypes (2).

Germinal centers (GC) are transient structures that form within secondary lymphoid tissues in response to T-cell dependent antigenic stimulation. GCs are initially established by highly proliferative centroblasts that form the GC dark zone and undergo immunoglobulin somatic hypermutation (3). After several rounds of division these cells migrate towards a region rich in T follicular helper cells (TFH) as non-dividing centrocytes, to form the GC light zone. B-cells with increased affinity for cognate antigen will receive T-cell help, which will enable them to either return to the DZ for more rounds of somatic hypermutation, or exit the GC reaction to become plasma cells or memory B-cells (4). GC B-cells undergo massive changes in their transcriptional, epigenetic and 3D architectural states, which is required for them to manifest their distinctive phenotype (5). Along these lines, conditional knockout of the ATPase subunit of the cohesin complex, Smc3, showed that cohesin dosage regulates B cell transit through GCs (6). *Smc3* haploinsufficient (*Smc3* ^wt/–^) mice display GC hyperplasia, with increased proliferation, accumulation of centrocytes and impairment of plasma cell differentiation. Chromosomal architecture analysis by Hi-C revealed that *Smc3* ^wt/–^ centrocytes have decreased long-range chromosomal interactions between enhancers and promoters, and reduced expression of tumor suppressor genes linked to lymphomagenesis in humans. Consistent with these findings, *Smc3* haploinsufficiency accelerated lymphomagenesis in mice engineered for constitutive expression of the Bcl6 oncoprotein, which drives formation of diffuse large B-cell lymphomas (DLBCLs) (6).

Cohesin complex mutations are common in human cancers (7) including myeloid malignancies (8–10). Curiously, although *Smc3* behaves as a haploinsufficient tumor suppressor in GC B-cells, it is rarely if ever affected by somatic mutations in patients with GC-derived lymphomas. Yet *SMC3* dosage may still be relevant to human GC derived lymphomas since it was shown that patients with low *SMC3* expression experience inferior clinical outcomes (6). Therefore, to gain insight into how *SMC3* dosage might contribute to malignant lymphoma phenotypes we explored its transcriptional, architectural and genomic effects in murine B-cell and lymphoma models with *Smc3* haploinsufficiency, with correlations to human DLBCL patients.

## Methods

### Conditional Smc3-Deficient Mice

The Research Animal Resource Center of the Weill Cornell Medical College approved all mouse procedures. The *Smc3* allele was deleted by targeting exon 4 in a construct obtained from the EUCOMM consortium [*Smc3* ^tm1a(EUCOMM)Wtsi^ (8)]. The generated mice (*Smc3* ^fl/fl^) were crossed to B6.129P2(Cg)-*Ighg1* ^tm1(cre)Cgn^/J mice (11) (*Cγ1* ^cre^; The Jackson Laboratory) to generate germinal center specific heterozygous deletion of *Smc3*. *Cγ1* ^cre/cre^;*Smc3* ^wt/–^ mice were further crossed to *Ighm* ^wt/tm1(Bcl6)Rdf^ mice [*IμBcl6* (12)].

### Induced Germinal Center B Cell Culture System

Induced GC B cell (iGCB) cultures were performed as reported elsewhere (13). Briefly, splenic CD43^–^ cells were co-cultured with irradiated 40LB cells (13) in the presence of 1 ng/mL IL-4. Four days after plating, iGCBs were incubated for 1 h in the presence of demecolcine 0.01 µg/ml, and iGCBs were separated by carefully collecting the cells in suspension and used in karyotyping analysis.

### Karyotyping Analysis

Induced GCB-like cells from culture systems were treated for 1 h with 0.01 µg/mL *N*-methyl-*N*-deacetyl-colchicine. Following 45 min incubation at 37°C, the cultures were resuspended in pre-warmed 0.075 M KCl, incubated for an additional 10 min at 37°C and fixed in methanol:acetic acid (3:1). The fixed cell suspension was then dropped onto slides, stained in 0.08 μg/ml DAPI in 2 × SSC for 5 min and mounted in antifade solution (Vectashield, Vector Labs). Metaphase spreads were captured using a Nikon Eclipse E800 epifluorescence microscope equipped with GenASI Cytogenetic suite (Applied Spectral Imaging). For each sample a minimum of 50 inverted DAPI-stained metaphases were fully karyotyped and analyzed.

### Flow Cytometry

Single-cell suspensions from mouse spleens and were stained using the following fluorescence-labeled anti-mouse antibodies: from BD Biosciences, FITC anti-CD38 (BD558813; clone 90; dilution 1:500), BV421 anti-CD95/Fas (BD562633; clone Jo2; dilution 1/500), PE-Cy7 anti-CD86 (BD560582; clone GL1; dilution 1:400), PE anti-CD184/CXCR4 (BD561734; 2B11; dilution 1:250); from BioLegend, APC-Cy7 anti-B220 (103224; clone RA3-6B2; dilution 1:750) and AlexaFluor647 anti-pSer139-H2AX (613407; clone 2F3, dilution 1:200). For internal markers, cells were fixed and permeabilized with the BD Cytofix/Cytoperm fixation/permeabilization solution kit (BD Biosciences). Data were acquired on a BD FACSCanto II flow cytometer (BD Biosciences) and analyzed using the FlowJo software package (BD Biosciences).

### Patient Data

For survival analysis we used publicly available gene expression data from 322 DLBCL patients from British Columbia Cancer Agency, BCCA (14). Additional analysis have been done in 243 patients from an NCI cohort (15). For univariable and multivariable Cox analysis, we used data from the British Columbia Cancer Agency cohort, and from publicly available gene expression data of 757 DLBCL patients, an independent cohort from our institution (16–19). All patient data used in this manuscript has been previously de-identified.

### Whole-Exome Sequencing and Identification of Somatic Variants

Genomic DNA from tumors was extracted from the mouse Smc3/Bcl6 or Bcl6 tumors and the germline tail (wild type) using DNeasy Blood Tissue kit (Qiagen). 1 µg of the genomic DNA was used to prepare the whole exome sequencing libraries with the Agilent SureSelect kit (SureSelect Mouse All Exon Kit). Using the NovaSeq6000 platform (Illumina), paired end sequencing was performed on the Smc3/Bcl6 (n=10) and Bcl6 tumors (n=5), and the wild type specimens (n=4). The average sequencing converge in the targeted regions was >40X except for one wild-type sample where the average coverage was 18X; this sample was excluded from further analysis. The whole exome sequencing reads were aligned to the Mouse reference genome GRCm38/mm10 using bwa mem and the PCR duplicates were marked and removed using Picard. The aligned and de-duplicated reads were then realigned around the indels, mates fixed and recalibrated to be used for downstream analysis. Somatic mutations were called using a consensus approach, where point mutations and indels were identified using Strelka2, MuTect and VarScan, and variants called by minimum two tools were retained for further analysis. Additional filtering steps excluded variants with total read depth < 30, number of reads supporting the variant < 5, tumor variant allele frequency (VAF) < 10% and germline VAF > 1%. The somatic mutations were annotated using the Variant Effect Predictor (VEP) and known mouse dbSNPs were filtered out while retaining only the missense, silent and truncating mutations. Copy number alterations were identified using the CNVkit. The percent genome altered (gain or loss) was calculated as the percentage of the copy number segments altered based on the size of the mouse genome. For the calculation of the altered segments, copy number segments with log2 ratio threshold of <-0.1 and >0.1 was used to quantify loss and gains, respectively. All statistical tests for significance were performed using the Wilcoxon rank sum test in R.

### Whole Genome Sequencing Analysis

Primary naïve B cells isolated from *Smc3* ^wt/wt^ (n=3) or *Smc3* ^wt/–^ were cultured ex vivo to produce iGCs as explained (13). Genomic DNA was used to produce whole genome sequencing libraries using the KAPA LTP Library Preparation kit following manufacturer’s directions. Sequencing was done in NextSeq500 instrument using a 75 bp single-read sequencing cell. We used TIGER (20) to infer DNA copy number values at 1Kb windows in mm10 coordinates. TIGER separates continuous and low-amplitude signals of DNA replication timing from the larger and sharper changes caused by copy number alterations. For genome-wide visualization of raw DNA copy number values, every 40 consecutive windows were merged. For DNA replication timing, outlier segments representing putative copy number alterations were filtered out by TIGER, and the remaining data was smoothed, normalized to units of standard deviation, and plotted.

### RNA Sequencing

mRNA-seq Library Preparation and RNA-seq libraries were prepared using the Illumina TruSeq RNA sample kits, according to the manufacturer. Libraries were validated using the Agilent Technologies 2100 Bioanalyzer and Quant-iT dsDNA HS Assay (Life Technologies) and 8–10 pM sequenced on HiSeq2000 sequencer. RNA-seq data was processed using the nf-core/rnaseq pipeline (v1.4.2) (21). Reads were aligned to mm10 and Gencode M12 (22) transcripts using STAR (v2.6.1) (23). Gene expression quantified by featureCounts (v1.6.4) (24) to counts and normalized to Transcripts per Million (TPM) (25). Differentially expressed genes between Smc3wt/– and Smc3wt/wt were identified using count data with a negative binomial model with the DESeq2 package (26). Pathway enrichment was calculated by using GSEA (27) and FGSEA (v1.14.0) (28) on the log_2_ fold change ranking results from DESeq2 output (26) with gene signature databases from literature, using murine and human orthologs of genes as necessary.

### Hi-C and Virtual 4C

1.5 × 10^6^ flow sorted mouse GC B cells from Cγ1^wt/cre^;Smc3^wt/wt^ (n=3) and Cγ1^wt/cre^;Smc3^wt/–^ (n=3) were fixed in 1% formaldehyde for 10 min. Fixation was quenched by the addition of 0.125 M glycine for 10 min. In situ Hi-C was performed as described (29). Briefly, nuclei were permeabilized and DNA was digested overnight with 100 U DpnII (New England BioLabs). The ends of the restriction fragments were labeled using biotin-14-dATP and ligated in 1 mL final volume. After reversal of crosslinks, ligated DNA was purified and sheared to a length of ~400 bp, at which point ligation junctions were pulled down with streptavidin beads, DNA fragments repaired, dA-tailed and Illumina adapters ligated. Library was produced by 6-10 cycles of PCR amplification. Sequencing was performed in a HiSeq2500 Illumina Sequencer, pair-end 50 bp, in the Weill Cornell Medicine Epigenomics Core.

All Hi-C data were processed using the hic-bench platform (30). In short, reads were aligned against the mouse genome (mm10) with bowtie (31) and multi-mapped, single-sided, duplicated, low quality and self-ligated reads were filtered with genomic-tools (32). Contact matrices were built with hic-bench at 20kb and 100kb resolution. Compartment analysis was performed with the c-score tool (33) at 100kb resolution, and A and B compartments were defined with the help of H3K27ac information. Compartment differences were defined as the difference in c-scores, called delta c-score. Loop analysis was performed with the mango loop calling approach (34), using a negative binomial test per diagonal in the 20kb resolution contact matrix, followed by multiple testing correction. Only loops with FDR<0.1 and CPM>30 were kept as significant loops. Differential loop analysis reported the log_2_ fold-change between CPM values per significant loop called in either sample. Protein-coding gene promoters and enhancer information were overlapped with all loop anchors, and promoter-enhancer loops were defined if one anchor holds at least one protein-coding gene promoter and the other anchor holds at least one enhancer. Virtual 4C analysis was performed based on the filtered reads. Filtered read pairs for which one read maps within +/– 10kb around the virtual viewpoint of the *Tet2* promoter (chr3:133,544,706) were extracted. Next, the genome was binned in successive overlapping windows of 20kb, and all adjacent windows are overlapping by 95% of their length (that is 19kb). We then added a count to all overlapping bins in which the second mapped read mate aligned. Read counts were then normalized to the total sequencing depth of the respective sample by edgeR reporting counts-per-million (CPM) per bin. Rad21 ChIP-seq in the CH12.LX mouse lymphoma cell line was downloaded from ENCODE (35, 36).

### Single-Cell RNA Sequencing

Splenic cells were sorted from *Smc3* ^wt/wt^ (n=6) and *Smc3* ^wt/–^ (n=3) mice 8 days after SRBC immunization. Sorted cells were subjected to single cell RNA-seq using the 10X Genomics Chromium platform. Library preparation for single cell 3’ RNA-seq v2, sequencing and post-processing of the raw data was performed at the Epigenomics Core at Weill Cornell Medicine. Libraries were prepared according to 10X Genomics specification and clustered on HiSeq4000. Sequencing data was processed with Cell Ranger from the 10X Genomics Cell Ranger Single Cell Software suite v3.0.2 (https://support.10xgenomics.com/single-cell-gene-expression/software/pipelines/latest/what-is-cell-ranger) using the manufacturer parameters to generate a sparse matrix file of features by barcodes. This sparse matrix data was then loaded into R (v4.0.2) using the R package Seurat (v4.0.0) (37). Additional wild-type 10X single-cell RNA-seq data was integrated with the Smc3 single-cell dataset to reduce batch effect. To identify genes and cells suitable for inclusion in the analysis, standard quality control was run to remove cells with few genes or an over representation of mitochondria reads. Data was then scaled and normalized. Linear dimensional reduction was performed by calculation of PCA from the most variable genes. Cells were then clustered using a resolution value of 0.5 and visualized by UMAP. Module scores were calculated using the AddModuleScore function with a control value of 5. Individual genes and gene signatures were projected and used to manually classify clusters. Centroblast (CB) and centrocyte (CC) cell clusters were identified using gene signatures defined by germinal center microarrays of DZ and LZ genes (38). Transitioning centroblast to centrocyte (CB → CC) clusters were classified by overlap of both DZ and LZ markers. The transitioning centrocyte to centroblast (Recycling) cluster was classified by a light zone DECP upregulated signature (39). Plasma cell (PC) clusters were identified using gene signatures from RNA-seq data (40), and the plasma blast (PB) cluster was identified as expressing c-Myc and S phase genes in addition to PC gene signatures. Prememory B cells (Pre-MBC) clusters were identified using transcriptional gene markers (41), and were subset into naive B cells (NB/Pre-MBC) and memory B cells (Pre-MBC/MBC) based on IgD^+^ gene expression and Ccr6 gene signatures respectively. Cell division signatures from RNA-seq were derived from Scharer et al. (42) data by determining significantly upregulated (padj < 0.05, log2FC > 1) genes between cells that underwent 8 cell divisions (D8) and express CD138 (D8 CD138^+^) or not (D8 CD138^–^), and cells that did not divide (division 0, D0) as assessed by the CTV fluorescence by flow cytometry (42). These signatures were then used to calculate module scores, project onto UMAP, and downstream analysis. RNA trajectory analysis was performed using Slingshot (v1.6.1). This package was used to create a pseudotime based on a combination of PCA 1 and 2 calculated by Seurat, using the cells identified as Centroblasts as the anchor point. Three lineages were generated (Lineage 1: CB → CC → MBC, Lineage 2: CB → Recycling, Lineage 3: CB → PC), and Lineage 3 was projected onto UMAP and used in downstream analysis. Pseudotime density plots were generated by cell cluster using the ggplot2 (v3.3.2) geom_density function. Pseudotime scatter plots were generated by genotype using the geom_point function.

### Quantitative RT-PCR

RNA was prepared by TRIzol extraction (Invitrogen). cDNA was prepared using the Verso cDNA synthesis kit (Thermo Fisher Scientific) and detected by Fast SYBR Green (Thermo Fisher Scientific) on a QuantStudio 6 Flex Real-Time PCR System (Thermo Fisher Scientific). We normalized gene expression to that of *Hprt1* and expressed values relative to control using the ΔΔCT method. Results were represented as fold expression with the s.d. for two series of triplicates. The following primers were used in qPCR experiments: Smc3_F, 5’-GGCTTCCGAAGTTACCGAGA-3’; Smc3_R, 5’-CAATCGCTGCTCTGGACG-3’; Tet2_F, 5’-TAGCTTTGCGTCAGTGGAGA-3’; Tet2_R, 5’-TAGGGATGGCTGGCTCAAAA-3’; Hprt1_F, 5′-AGGACCTCTCGAAGTGTTGG-3′; Hprt1_R, 5′-TTGCAGATTCAACTTGCGCT-3′.

### Quantification and Statistical Analysis

The overall survival of DLBCL patients was estimated by Kaplan-Meier method. The mRNA expression levels of genes in the Smc3_*vs*_WT:CC_UP_logFC_0.56 gene signature [human orthologs: *H1F0*, *RP1L1*, *GSTT2B*, *GSTT2*, *THYN1*, *ALAD*, *IRAK1BP1*, *RANBP17*, *UBE2C*, *RET*, *GNB4*, *USP2*, *MFGE8*, *LGALS1*, *EMP2*, *TMED6*, *GCSAM*, *BFSP2*, *MYL4*, *GNAZ*, *TBXA2R*, *CPNE5*, *LRRC49*, *CCNB2*, *PAFAH1B3*, *CDC20*, *SCCPDH*, *AVIL*, *PI4KB*, *SSR2*, *CDKN3*, *NREP*, *TMOD4* (6)] were used for unsupervised hierarchical clustering on DLBCL patient cohorts. The differences of overall survival between two resulting clusters were tested by log-rank test. The univariable and multivariable Cox proportional hazard regression were also used to confirm the findings, while adjusting for age, sex and subtype. Statistical analyses were performed in statistical software R Version 3.6.0 (R Foundation for Statistical Computing, Vienna, Austria).

## Results

### Aberrant Transcriptional Programming in *Smc3* Haploinsufficient Lymphomas

**Figure 1:**
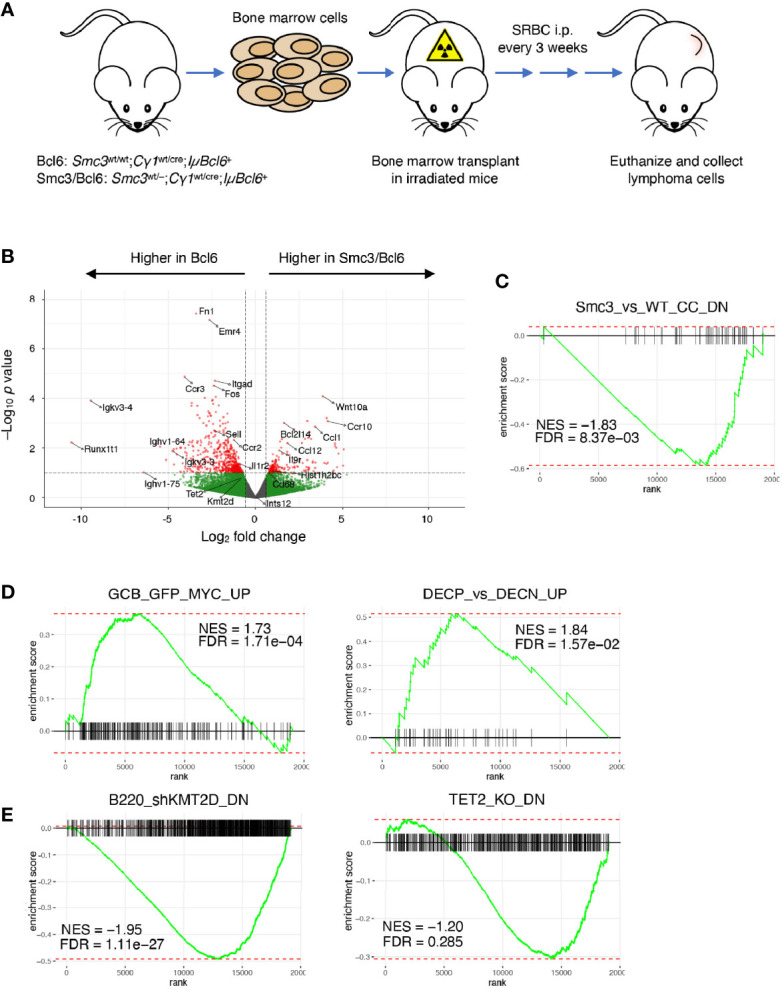
Aberrant gene expression program in Smc3 haploinsufficient tumors. **(A)** Development and experimental design for study of lymphomas in Bcl6 and Smc3/Bcl6 mice, where conditional heterozygous deletion of Smc3 was directed towards B-cells entering the GC reaction by crossing to the Cγ1-Cre strain. **(B)** Volcano plot showing differentially expressed genes in Bcl6 and Smc3/Bcl6 tumor cell RNA-sequencing. **(C–E)** Gene set enrichment analysis plots in Bcl6 *vs* Smc3/Bcl6 RNA-sequencing. Smc3_*vs*_WT_CC_DN (6), DECP_*vs*_DECN_UP, GCB_GFP_MYC_UP (39), B220_shKMT2D_DN (43) and TET2_KO_DN (44).

*Smc3* haploinsufficiency drives accelerated lymphomagenesis in *IµBcl6* transgenic mice (6). To explore whether this aggressive phenotype was linked to aberrant transcriptional programming, we performed RNA-seq from mesenteric lymph node lymphoma cells from the *Smc3* ^wt/wt^;*Cγ1* ^wt/cre^;*IµBcl6* (Bcl6) and *Smc3* ^wt/–^;*Cγ1* ^wt/cre^;*IµBcl6* (Smc3/Bcl6) mice, verifying the expected reduced expression of *Smc3* (**Figure 1A**, **Supplementary Table 1**, and **Supplementary Figure 1A**). Unsupervised analyses did not yield strong differences between these lymphomas (**Supplementary Figures 1B, C**), and no difference in other cohesin subunit or related genes (**Supplementary Figure 1D**). However, naturally occurring primary lymphomas are often highly heterogeneous including in the context of *IuBcl6* mice (12, 45, 46), which might interfere with our ability to appreciate changes in gene expression. Along these lines, a supervised analysis indeed revealed only subtle differences in the transcriptional programs of Bcl6 *versus* Smc3/Bcl6 mice with 199 genes upregulated and 537 genes downregulated in Smc3/Bcl6 tumors (pval < 0.1, |log2FC| > 0.56 used, **Figure 1B**), that were not captured using higher stringency parameters. In spite of this there was evident perturbation of transcriptional programming in these tumors, as noted by performing GSEA analysis, which revealed significant down regulation of genes that were previously shown to be repressed in *Smc3* ^wt/–^ centrocytes (6) (**Figure 1C**). More critically and consistent with the observed aggressive tumor phenotype, Smc3/Bcl6 lymphomas featured induction of canonical GC-associated MYC target gene sets (**Figure 1D**). We also found evidence of tumor suppressor effects, such as negative enrichment for genes down regulated in *Kmt2d* or *Tet2* deficient GCs (**Figure 1E**). Both of these genes are tumor suppressors in human DLBCLs (47), and *Tet2* loss of function was also shown to induce lymphomagenesis in *IuBcl6* mice (44). Overall these transcriptional perturbations similar to those caused by *Smc3* haploinsufficiency in GC B-cells, suggest that persistence of these effects contributes to its role in lymphomagenesis.

### Cohesin Haploinsufficiency Induces Loss of Tumor Suppressor Gene Promoter-Enhancer Interactions

**Figure 2:**
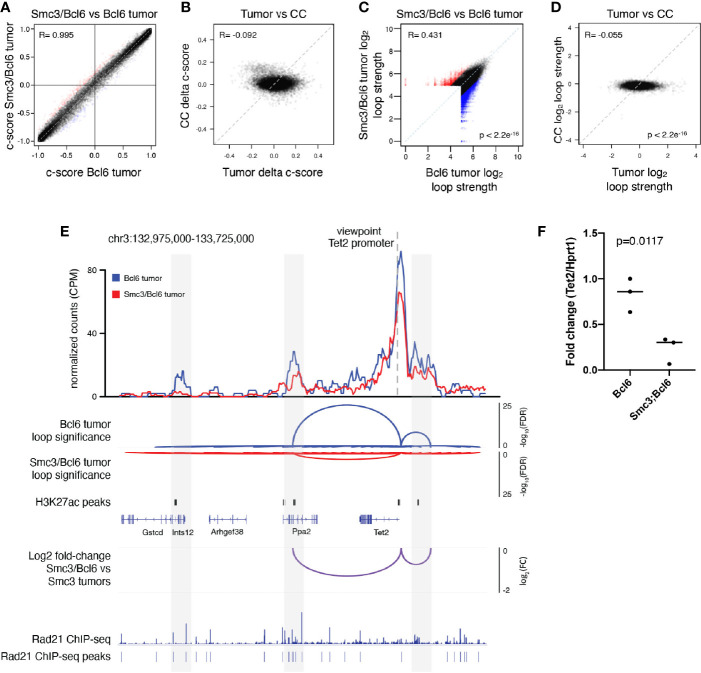
Cohesin haploinsufficiency induces loss of interactivity of promoter-enhancers at tumor suppressor genes. **(A)** Correlation plot between the compartment c-scores of Smc3/Bcl6 *versus* Bcl6 tumor cells at 100kb resolution. **(B)** Correlation plot for the change in compartment c-scores of Smc3/Bcl6 *versus* Bcl6 tumor cells and the change in compartment c-scores of *Smc3^wt^* ^/–^ *versus Smc3^wt^* ^/wt^ centrocytes (CC) at 100kb resolution. **(C)** Correlation plot for the log_2_ normalized loop interactivity of Smc3/Bcl6 *versus* Bcl6 tumor cells at 20kb resolution. **(D)** Correlation plot for the log_2_ fold change of normalized loop interactivity of Smc3/Bcl6 *versus* Bcl6 tumor cells and the log_2_ fold change of normalized loop interactivity of *Smc3* ^wt/–^ *versus Smc3* ^wt/wt^ centrocytes (CC) at 20kb resolution. **(E)** Virtual 4C analysis showing normalized interactions with the *Tet2* promoter for Bcl6 tumors (blue line) and Smc3/Bcl6 tumors (red line) at 20kb resolution. Loop calling significance following the mango approach are shown for Bcl6 and Smc3/Bcl6 tumors with –log_10_(FDR). Enhancers were defined as H3K27Ac peaks mapped in germinal center B cells by Mint-ChIP. Rad21 ChIP-seq was performed in the mouse lymphoma cell line CH12.LX (35). The differences between normalized interactions with the *Tet2* promoter are shown as log_2_ fold-change between Bcl6 and Smc3/Bcl6 tumors. **(F)** RT-qPCR for *Tet2* mRNA in Bcl6 (n=3) and Smc3/Bcl6 (n=3) tumors, normalized to *Hprt1* mRNA expression.

In order to explore whether these changes in gene expression or other aspects of the malignant phenotype might be linked to 3D architectural effects, we performed *in situ* Hi-C in lymphoma cells collected from involved mesenteric lymph node tumors of moribund Bcl6 (n=3) and Smc3/Bcl6 (n=3) mice. Hi-C contact maps revealed little difference globally between Smc3/Bcl6 *vs* Bcl6 tumor interactivity profiles (**Supplementary Figure 2A**). This is consistent with genomic chromatin compartmentalization being independent of cohesin subunit dose, as previously reported (48). Indeed further examination of chromatin compartment distribution in Smc3/Bcl6 *vs* Bcl6 and tumor cells showed very little difference between these genotypes (**Figure 2A** and **Supplementary Figure 2B**). In contrast, there were significant compartment changes among these lymphomas as compared to normal centrocytes (**Figure 2B**). Hence aberrant chromatin compartmentalization in these lymphomas must occur through a cohesin independent manner as well. Focusing instead on differential chromatin interactivity, we found a significant bias towards reduction in chromatin loop strength in Smc3/Bcl6 *vs* Bcl6 lymphomas (**Figure 2C**). There was also significant difference in loop strength when comparing all murine lymphomas to normal centrocytes (**Figure 2D** **)**. Examining differential chromatin interactions in more detail revealed reduction in loop strength of enhancer-promoter loops as well as other chromatin interactions (**Supplementary Figure 2C**). Among genes with reduced enhancer-promoter loops were known tumor suppressors such as *Tet2*, *Dusp4*, as well as MHC class II genes. Conversely genes such as *Cdk6, Btk* and *Irak1* were among those with stronger enhancer to promoter looping. Decreased loop interactivity in Smc3/Bcl6 *versus* Bcl6 tumors was also appreciated by performing aggregate peak analysis (**Supplementary Figure 2D**).

The reduction of *Tet2* enhancer promoter loop strength observed in this global analysis prompted us to look more closely at this tumor suppressor gene. For this we performed virtual 4C analysis using our Hi-C data (**Figure 2E**), anchored at the *Tet2* promoter and observed marked reduction of its interactivity with upstream and downstream regions (**Figure 2E**). These sites overlapped with putative enhancers defined by the presence of H3K27Ac peaks identified by Mint-ChIP-seq from GC B-cells (6) and with cohesin subunit Rad21ChIP-seq peaks in murine CHX.12 lymphoma cells (35). Strikingly, this reduction in *Tet2* promoter to enhancer looping was associated with reduced abundance of *Tet2* mRNA in Smc3/Bcl6 *vs* Bcl6 lymphomas from qPCR experiments performed in independent lymphoma specimens (**Figure 2F**). Tumor suppressor genes *Kmt2d* and *Dusp4* showed similar loss of interactivity of their promoters with putative H3K27Ac rich loci (**Supplementary Figures 2E, F**) in Smc3/Bcl6 tumors. Taken together with our transcriptional profiling showing enrichment for *Tet2* and Kmt2d deficient signatures, these data suggest that reduced levels of *Smc3* in lymphomas impairs expression and functionality of tumor suppressor genes through disruption of enhancer-promoter interactions.

### *Smc3* Haploinsufficiency Specifically Impairs Terminal Steps of Plasma Cell Differentiation

**Figure 3:**
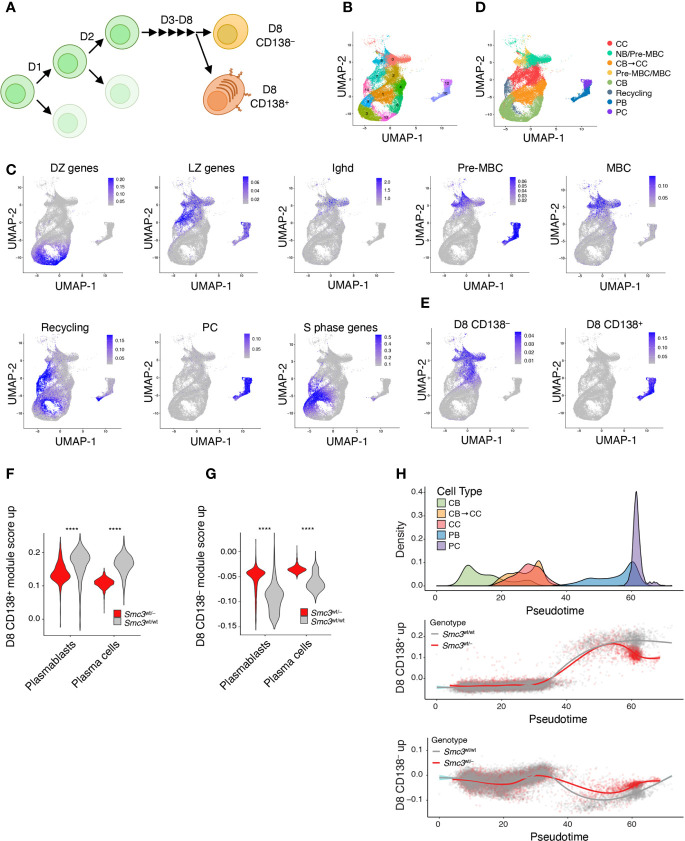
Smc3 haploinsufficient cells undergo proliferation burst but fail to differentiate into plasma cells. **(A)** Scheme depicting mature B cell differentiation leading to the plasma cell phenotype. **(B)** Uniform Manifold Approximation and Projection (UMAP) applied to single cell RNA-seq populations of germinal center and post germinal center populations. **(C)** UMAP projections of gene expression cell signatures used to classify clusters. **(D)** Applying previously defined gene expression signatures, Seurat clusters were manually defined as centrocyte (CC), pre-memory B cells (Pre-MBC), transitioning centroblast to centrocyte (CB → CC), transitioning centrocyte to centroblast (Recycling), memory B cells (MBC), plasmablast (PB), and plasma cells (PC). **(E)** UMAP showing the projections of *in vivo* LPS-stimulated CFSE stained B cells that divided 8 times expressing CD138 (D8 CD138^+^) or not (D8 CD138^–^). **(F)** Violin plots showing expression levels of D8 CD138^+^ for PB and PC clusters. **(G)** Violin plots showing expression levels of D8 CD138^–^ for PB and PC clusters. **(H)** Cell densities for pseudotime lineage (CB → CB to CC → CC → PB → PC, top plot) and scatter plots of cells by genotype across pseudotime lineage expressing D8 CD138^+^ (middle panel) or D8 CD138^–^ (lower panel) profiles. ****p < 0.00001.

Conditional deletion of *Smc3* in GC B-cells results in impaired plasma cell differentiation (6). Our data shown above suggest that this effect persists in *Smc3* haploinsufficient lymphomas, pointing to plasma cell differentiation as a key vulnerability for malignant transformation. However, this is a step-wise and complex process, and the precise point in plasma cell differentiation where the *Smc3* function becomes critical is not known. Along these lines, Scharer et al. revealed that mature B cells induced to form plasma cells undergo ~8 cell divisions prior to acquiring the full plasma cell phenotype (42). Scharer et al. performed RNA-seq at sequential cell divisions in selected populations based on cell cycle dye exclusion and CD138 staining, as well as single cell RNA-seq of activated B cells to precisely map plasma cell differentiation trajectory. This trajectory was complex and included a critical cell fate decision that took place upon the last (8^th^) cell division, whereupon B-cells either committed to the final plasma cell phenotype or remained in a less defined B-cell state (**Figure 3A**) (42).

To define the point along this trajectory that was specifically dependent on *Smc3* dosage, we performed single cell RNA-seq in *Smc3* ^wt/–^ and *Smc3* ^wt/wt^ GC B cells. We defined cell clusters by unsupervised analysis using Seurat and then projected canonical GC and post-GC related signatures from centroblasts (DZ), centrocytes (LZ), plasma cells (PC), memory B-cells (MB), and MYC^+^ GC B-cells (selected by T-cell help) onto these transcriptional profiles. This allowed us to assign clusters of cells to these various cell subpopulations (**Figures 3B–D** **)**. Plasma cells were further subdivided into plasmablasts *vs* plasma cells based on the former expressing MYC-associated and S phase genes (**Figure 3C**). Other cell clusters were assigned as intermediate between DZ and LZ, possibly reflecting cells transitioning from DZ to LZ. In addition to MB cells, we identified cell clusters enriching for a mixture of cells with pre-MB signature, with IgD^+^ naïve B-cells.

We then projected the RNA-seq signatures derived from the data from Scharer et al. (42), by comparing their division 8 (D8) CD138^+^ or D8 CD138^–^ profiles with those from baseline (day 0) mature B-cells (**Figure 3E**). D8 CD138^+^ cells largely overlapped with plasmablast and plasma cells, whereas D8 CD138^–^ overlapped with centrocytes and memory/pre-memory B cells (**Figure 3E**). Examining the plasmablast and plasma cell populations from our single cell RNA-seq dataset we observed depletion of D8 CD138^+^ signature gene scores among *Smc3* ^wt/–^ cells **(** **Figure 3F** **)**, whereas in contrast these cells scored more highly for D8 CD138^–^ signature gene expression **(** **Figure 3G** **)**. Performing pseudotime analysis to distribute cells according to their differentiation state from centroblast towards plasma cell transcriptional programming (**Supplementary Figure 3**), we observed impaired acquisition of the D8 CD138^+^ signature among *Smc3* ^wt/–^ haploinsufficient plasma cells, suggesting defective engagement of the late-stage plasma cell commitment program (**Figure 3H**). In contrast, the D8 CD138^–^ signature scored higher among *Smc3* ^wt/–^ plasma cells, suggesting a strong bias away from the final stages of plasma cell commitment and preferential maintenance of B-cell transcriptional signatures. This branching point may represent a particularly vulnerable architectural checkpoint for malignant transformation.

### *Smc3* Haploinsufficiency Increase DNA Damage in Germinal Center B Cells

**Figure 4:**
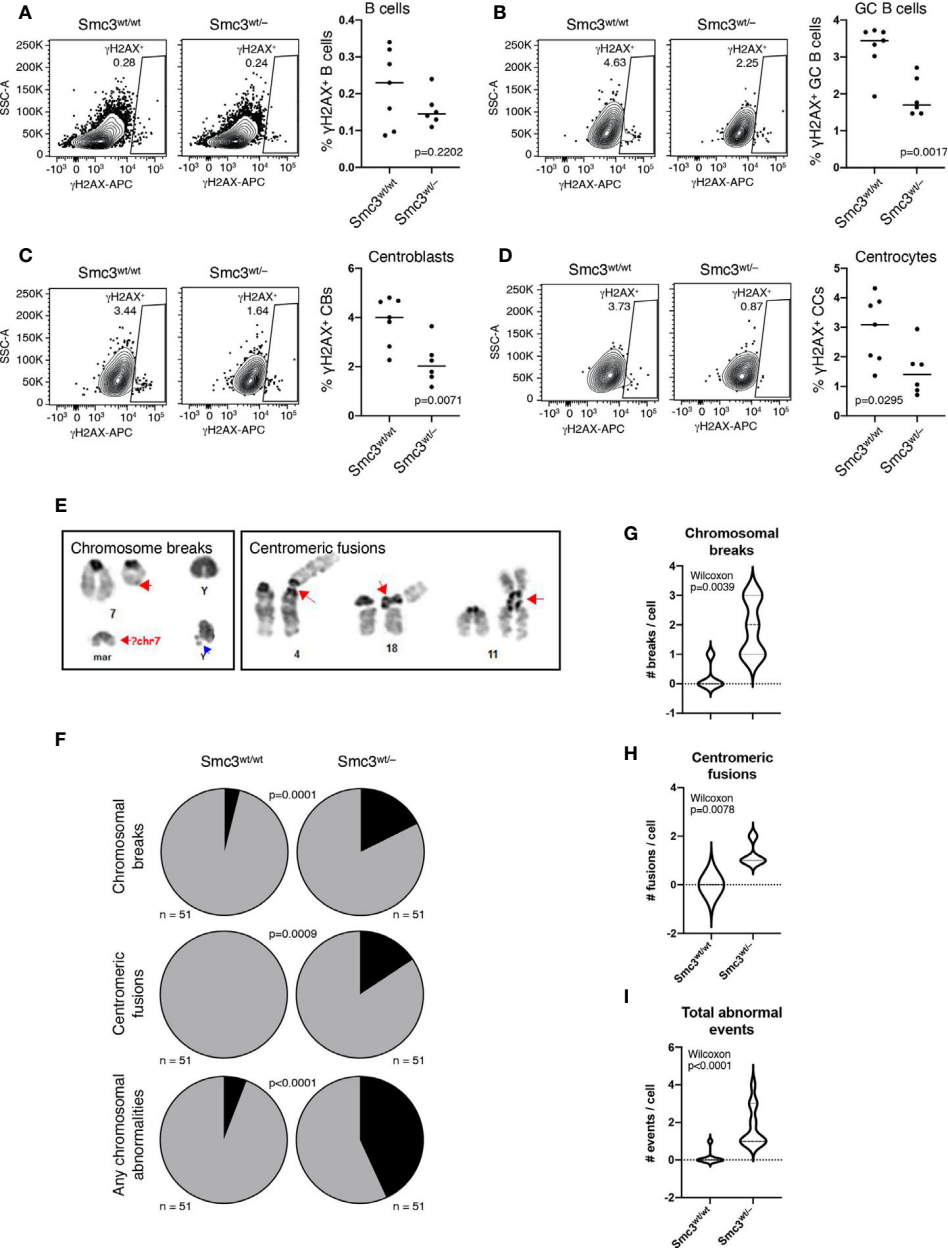
Smc3 haploinsufficiency increases DNA damage in germinal center B cells. **(A–D)** Gating strategy used to detect phospho-γH2AX by flow cytometry (left) and quantification (right) in B cells **(A)**, germinal centers **(B)**, centroblasts **(C)**, and centrocytes **(D)**. **(E)** Identification and classification of chromosomal aberrations in induced GC B cells (iGC). **(F)** Quantification of the frequency of chromosomal breaks and centromeric fusions in iGCs of Smc3^wt/wt^ (n=3) and Smc3^wt/–^ (n=3) mice, and **(G),** quantification of chromosomal breaks, **(H),** centromeric fusions per cell, or **(I)** total events per cell. Experiment shown is a representative one from 3 performed. P values calculated using a binomial test for expected *versus* observed frequencies **(F)** and Wilcoxon rank test for count distribution **(G-I)**.

Given that GC B-cells are exposed to considerable DNA damage stress (49, 50) and cohesin complex is reported to play important roles in DNA damage response (51, 52), we wondered whether *Smc3* haploinsufficiency might also contribute to lymphomagenesis through accumulation of DNA damage. Phosphorylation of Ser-139 residue of histone H2AX, forming γH2AX, is an early cellular response to the induction of DNA double-strand breaks that has been shown to be dependent on the loop extrusion activity of cohesin (53). We therefore used flow cytometry to measure γH2AX staining in total splenic B cells (live B220^+^ cells) or GC B-cells (B220^+^FAS^+^CD38^–^) from *Smc3* ^wt/–^ or *Smc3* ^wt/wt^ mice, eight days after immunization (**Supplementary Figures 4A, B**). Notably, although we did not observe differences in total live B cells (**Figure 4A**), we observed a significant reduction of γH2AX^+^ staining in *Smc3* ^wt/–^ GC B-cells (**Figure 4B**). Notably the reduced abundance of γH2AX was evident in both centroblasts (B220^+^FAS^+^CD38^–^CXCR4^+^CD86^–^) and centrocytes (B220^+^FAS^+^CD38^–^CXCR4^–^CD86^+^, **Figures 4C, D**). *Smc3* haploinsufficiency did not result in differential apoptosis in GC B cells (6). The lack of apoptosis along with the reduced γH2AX suggested that there might be impaired DNA damage detection in Smc3 haploinsufficient cells.

For more direct assessment of DNA damage, we performed karyotype analysis in proliferating *Smc3* ^wt/–^ *and Smc3* ^wt/wt^ GC B cells. Since obtaining abundant actively proliferating GC B cells from murine lymphoid tissue is not possible, we instead used the induced GC B cell (i-GCB) co-culture system, to produce high numbers of proliferating iGCB cells (**Supplementary Figure 4C**) (13). Karyotyping analysis was used to identify chromosomal aberrations (**Figure 4E**). Examining metaphase spreads from these cells revealed significantly higher abundance of lesions such as centromeric fusions or chromosomal breaks in *Smc3^wt/–^* GC B-cells (**Figures 4F–I** and **Supplementary Figure 4D**). Notably, centromeric fusions were completely absent from wild type iGCB cells, suggesting these are highly cohesin dose dependent. Whole genome sequencing in i-GC failed to demonstrate detectable structural lesions or differences in replication fork usage or activation (**Supplementary Figures 5A, B**).

**Figure 5:**
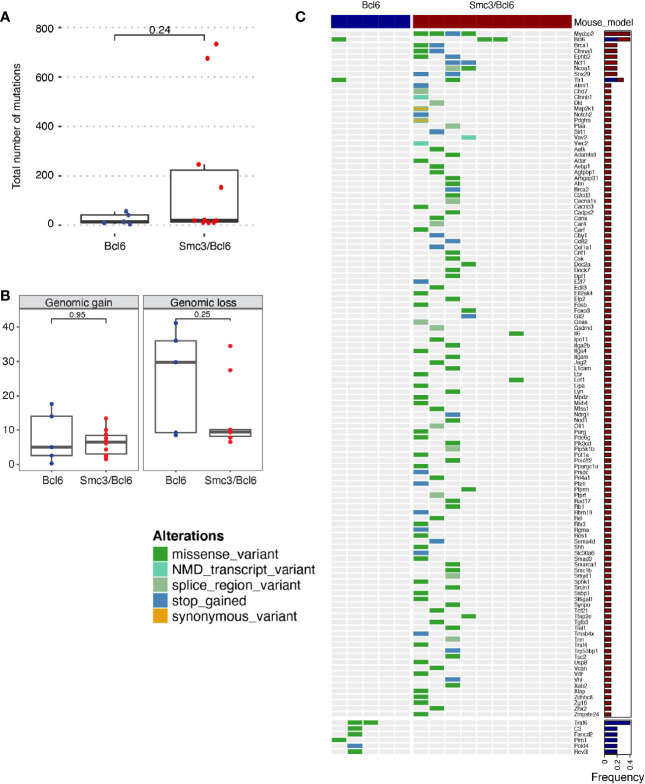
Mutational analysis of Smc3/Bcl6 tumors. **(A, B)** Mutational burden of Bcl6 and Smc3/Bcl6 tumors assessed by exon capture analysis. **(A)** number of mutations per tumor. **(B)** Genomic gain and loss in Bcl6 and Smc3/Bcl6 tumors. **(C)** Oncoprint depicting AID-induced mutations in 125 non-immunoglobulin genes in Bcl6 (n=5) and Smc3/Bcl6 (n=10) tumors. Mutation frequency is shown in the right bar plot for Bcl6 (blue) and Smc3/Bcl6 (red). Mutations were classified as missense mutations, nonsense-mediated decay transcript variants, splice regions variants, stop gained, or synonymous variants, as indicated by the color key on the left of the oncoprint.

These observations prompted us to perform exome capture for mutation profiling in Smc3/Bcl6 *vs* Bcl6 lymphoma cells, obtained from lymphoid tissues of moribund animals. Although Smc3/Bcl6 tumors showed higher variability in the total numbers of somatic mutations, these were not significantly different than tumor cells from the Bcl6 (Wilcoxon p=0.24, **Figure 5A**). Copy number gains and losses quantified as the percent mouse genome altered were also not significantly different between the Smc3/Bcl6 and the Bcl6 mouse models (**Figure 5B**). Activation induced cytosine deaminase (AICDA) is the main source of mutations in germinal center B-cells during the process of somatic hypermutation (3). We thus analyzed the mutation frequency of 125 off-target genes (i.e. non-immunoglobulin, **Supplementary Table 2**) in Bcl6 and Smc3/Bcl6 tumors. Interestingly, we found that only 8 genes were mutated in at least one Bcl6 tumor, while 119 of them were mutated in at least one Smc3/Bcl6 tumor. The identity of those genes was also different between the tumors, with *Traf6* and *Pim1* being amongst the most frequently mutated genes in Bcl6 tumors, and *Mycbp2* and *Brca1* amongst Smc3/Bcl6 tumors (**Figure 5C** and **Supplementary Table 2**). Overall, the lack of a clear gain in structural genomic variants in Smc3/Bcl6 lymphomas suggests that the types of lesions induced by Smc3 deficiency in GC B-cells may not yield efficient trajectories for malignant transformation, although the reduced DNA damage sensing may lead to accumulation of mutations in AICDA off-target genes.

### Decreased Cohesin Levels Predict Poor Survival in DLBCL Patients

**Figure 6:**
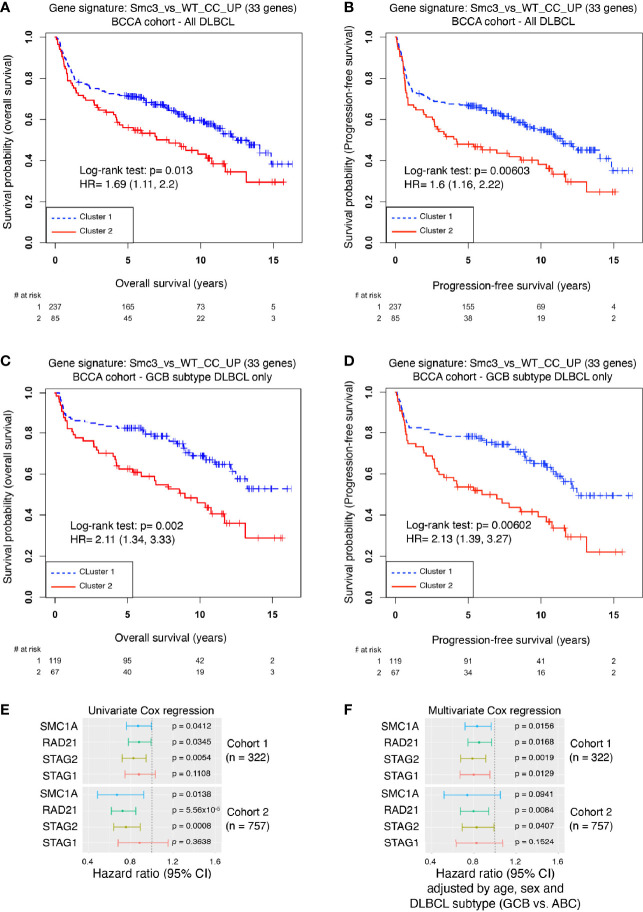
Decreased cohesin levels predict poor survival in DLBCL patients. **(A)** Kaplan-Meier overall survival curves for DLBCL patients (n=322) in BCCA cohort clustered with the *Smc3* haploinsufficient gene signature (6). **(B)** Kaplan-Meier progression-free survival curves for DLBCL patients (n=322) in BCCA cohort clustered with the *Smc3* haploinsufficient gene signature (6). **(C)** Kaplan-Meier overall survival curves for GCB-subtype DLBCL patients (n=186) in cluster 1 and 2. **(D)** Kaplan-Meier progression-free survival curves for GCB-subtype DLBCL patients (n=186) in cluster 1 and 2. **(E)** Univariate Cox regression analysis, and **(F)** multivariate Cox regression analysis were performed in two cohorts of DLBCL patients (cohort 1, n = 322 individuals; cohort 2, n = 757 individuals). In both cases, *SMC1A*, *RAD21*, *STAG2* and *STAG1* expression levels were used as a continuous variable. Multivariate analysis was adjusted by age, sex and DLBCL subtype of the individual. Error bars represent 95% confidence intervals of the hazard ratio.

The enrichment of *Smc3* ^wt/–^ centrocyte transcriptional signature in accelerated lymphomas induced by *Smc3* haploinsufficiency, prompted us to explore whether these profiles are linked to clinical outcome DLBCL patients. Examining the RNA-seq profiles of 322 newly diagnosed DLBCL patients, we performed unsupervised clustering to define DLBCL patient clusters with high and low expression of human ortholog genes that are repressed in *Smc3* ^+/–^ centrocytes (**Supplementary Figure 6A**). Cluster 1 contained 237 DLBCL patients and cluster 2 contained 85 DLBCL patients. Remarkably, patients in cluster 2 manifested significantly inferior overall survival (Log-rank test p=0.013, HR=1.69, 95% CI=1.11-2.2, **Figure 6A**) and inferior progression-free survival (Log-rank test: p=0.006, HR=1.6, 95% CI=1.16-2.22, **Figure 6B**) compared to those in cluster 1. To determine whether *Smc3* haploinsufficiency signature was associated with reduced expression of cohesin complex genes we examined the relative expression of *SMC3*, *SMC1A*, *RAD21*, *STAG1* and *STAG2* in our DLBCL patient cohort. Strikingly, all five of these genes were significantly reduced among the patients in cluster 2 (**Supplementary Figure 6B**). When DLBCL tumors were classified according to their gene expression profiles as belonging to the germinal center B cell-like subtype (GCB, n=186) or activated B cell-like subtype (ABCs, n=108), we observed that the cohesin low cluster 2 still displayed decreased overall survival (Log-rank test p= 0.002, HR=2.11, 95% CI=1.34-3.33, **Figure 6C** **)**, and decreased progression-free survival (Log-rank test p=0.006, HR=2.13, 95% CI=1.39-3.27, **Figure 6D**) in the GCB subtype, but not among the ABC-DLBCLs (overall survival Log-rank test p=0.86, HR=1.05, 95% CI=0.63-1.72, **Supplementary Figure 6C**, and progression-free survival Log-rank test p=0.931, HR=0.98, 95% CI=0.6-1.59, **Supplementary Figure 6D**).

We validated these findings in an independent cohort of 243 DLBCL patients (15), where unsupervised clustering using the *Smc3* haploinsufficient gene signature defined two clusters, of 156 and 87 patients, respectively (**Supplementary Figure 6E**). In striking similarity, cluster 2 displayed decreased expression of all five cohesin core subunits (**Supplementary Figure 6F**) and a significantly shorter overall survival (Log-rank test p=0.0174, HR=1.64, 95% CI=1.10-2.45, **Supplementary Figure 6G**) compared to cluster 1.

Consistent with our findings, lower abundance of *Smc3* mRNA was shown to be associated with worse clinical outcome (6), but our data suggest a broader association of clinical outcomes with cohesin subunit expression. We therefore performed univariate Cox regression for cohesin subunits *SMC1A*, *RAD21*, *STAG1* and *STAG2*, and found a similar inverse correlation with overall survival across two independent cohorts of 322 and 757 DLBCL patients, with the exception of *STAG1* (**Figure 6E**). This effect was still observed in multivariate Cox analysis that include age, sex and DLBCL subtype (**Figure 6F**). These results strongly link reduced cohesin dosage with more aggressive disease among DLBCL patients, in line with observations of lymphomagenesis in *Smc3* haploinsufficient mice (6).

## Discussion

**Figure 7:**
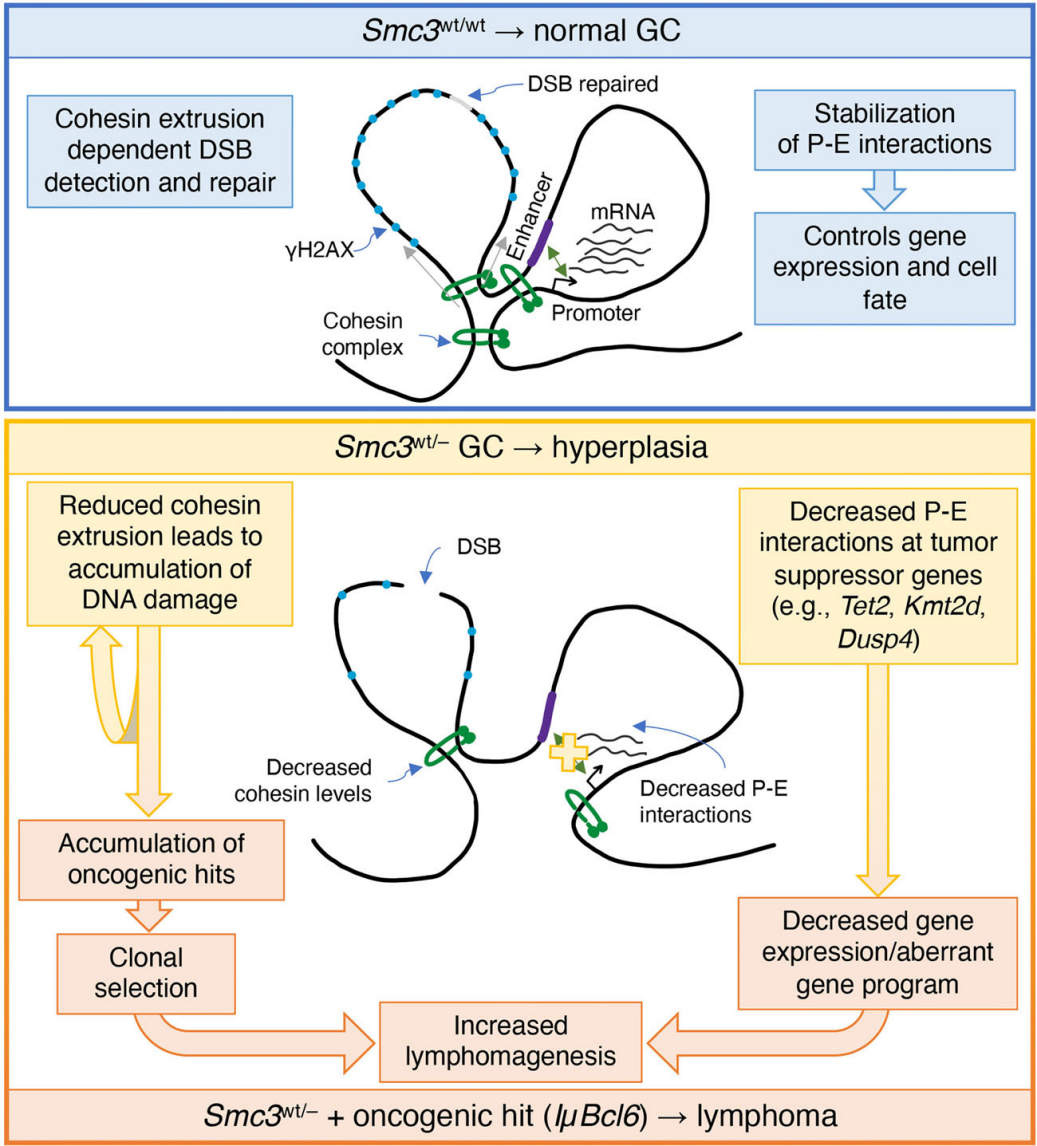
Model of cohesin haploinsufficiency induced lymphomagenesis. Biallelic dosage of the *Smc3* cohesin subunit enables promoter-enhancer (P-E) interactions of critical genes for cell identity and cell fate. In addition, the extrusion function of cohesin plays an important role in detection of double strand breaks (DSB) and establishment of phosphorylated histone H2AX (γH2AX). In *Smc3* haploinsufficient GCs, decreased promoter-enhancer interactions in tumor suppressor genes such as *Tet2*, *Kmt2d* and *Dusp4*, result in decreased gene expression and/or alteration of the gene program. Defective detection of DSB leads to accumulation of chromosomal aberrations that are mechanistically linked to reduced abundance of Smc3 protein and hence fewer cohesin loop extrusion complexes, both of which may play a role in lymphomagenesis.

Recent pan-cancer studies have shown that cohesin and its regulators are among the most frequently mutated genes in cancer. Mutations in genes encoding cohesin subunits were first reported in colorectal cancer (54), and later in glioblastoma, Ewing sarcoma and melanoma (55). Chromosome missegregation has been suggested as a mechanism of cohesin dysfunction to tumorigenesis. Yet sequencing of acute myeloid leukemia (AML) patient specimens revealed the presence of recurrent mutations in all four core cohesin subunits but not associated with cytogenetic abnormalities (56). The correlation between STAG2 mutations and aneuploidy in bladder cancer is also unclear (57, 58). Even though somatic mutations of core cohesin genes in GC derived lymphomas are exceptionally rare, it was previously shown that *Smc3* could still function as a tumor suppressor in these cells (6). Herein was explored potential mechanisms through which this might occur and examined this from both the genomic stability and transcriptional regulatory standpoints (**Figure 7**).

Notably we did observe chromosomal structural aberrances in cohesin haploinsufficient GC cells, in contrast to what has been reported in myeloid cells (59). This might be explained by the fact that GCB cells are already at increased genotoxic stress compared to other cells types. For example, it is well established that the critical GCB transcription factor BCL6 represses checkpoint and DNA damage response genes (49, 50). Therefore, it is possible in this context that DNA damage due to reduced cohesin dosage is not properly sensed or repaired, tipping the balance towards accumulation of DNA damage. In spite of this, we did not observe increased abundance of DNA damage in *Smc3* haploinsufficient murine lymphomas. Perhaps this may be due to cells experiencing major chromosomal structural aberrancies being negatively selected during the transformation process. Nonetheless, taking together the apparent impairment in DNA damage sensing that we observed in Smc3^wt/–^ GC B-cells and more frequent mutations in AICDA off-target genes in Smc3/Bcl6 lymphomas does suggest a potential genetic contribution of Smc3 deficiency to lymphomagenesis, pointing to the need for further investigation into this possibility. Along these lines, a recent publication revealed a role for the cohesin complex during DNA damage and γH2AX mark deposition (53). According to that model, cohesin complex loop-extrusion activity plays a critical role in detection of double strand breaks and topologically associating domains are the functional units of the DNA damage response, being instrumental for the correct establishment of γH2AX–53BP1 chromatin domains in a manner that involves one-sided cohesin-mediated loop extrusion on both sides of the double strand break. The authors proposed that H2AX-containing nucleosomes are rapidly phosphorylated as they actively pass by double strand breaks-anchored cohesin. Here, we speculate that cohesin haploinsufficiency attenuates detection of double strand breaks. This would explain both the decreased levels of γH2AX and increased chromosomal aberrations observed in Smc3^wt/–^ GC B-cells.

On the other hand, *Smc3* haploinsufficient lymphomas did manifest transcriptional and architectural perturbations consistent with those observed in *Smc3* haploinsufficient centrocytes. This includes repression of genes that are also aberrantly repressed by loss of function of two DLBCL epigenetic tumor suppressor genes *TET2* and *KMT2D*. *Tet2* normally mediates enhancer cytosine hydroxymethylation whereas *Kmt2d* mediates enhancer H3K4 mono and demethylation (60, 61). Loss of function of these genes leads to impaired enhancer function with repression of the respective genes and accelerated lymphomagenesis in mice (43, 44). This is reminiscent of and consistent with the impaired enhancer-promoter interactions that we observe by Hi-C in *Smc3* ^wt/–^ murine lymphomas. The phenotype of *Tet2* ^–/–^ GCs is especially similar to that of *Smc3* ^wt/–^ and *Tet2* deficiency also cooperates with *Bcl6* to induce accelerated lymphomagenesis (44). The finding that the *Tet2* gene itself showed impaired connectivity with upstream and downstream enhancers and reduced expression in *Smc3* ^wt/–^ murine lymphomas further underlines the potential mechanistic and biological links between *Tet2* and cohesin complex in GC lymphomagenesis. Along these lines, it is notable that *Smc3* ^wt/–^ signature is linked to reduced expression of cohesin complex genes and is most clinically significant in GCB-subtype DLBCL, where *Tet2* and *Kmt2d* loss of function are most clearly deleterious (44, 62). Although tumors derived from *Smc3* haploinsufficient B cells display a *Kmt2d* loss of function-like transcriptional profile, we did not detect consistent downregulation of *Kmt2d* mRNA itself in tumor cells. Whether the transcriptional profile observed is due to an earlier downregulation of *Kmt2d* and epigenetic maintenance of the aberrant transcriptional status or if it is simply due to overlap with *Tet2* loss of function signature remains unknown. Taken together, these findings, suggest that the oncogenic impact of cohesin loss of function in GC B-cells is mainly due its transcriptional and architectural effect related to gene enhancers, and not to genomic instability. It is interesting to speculate to what extent cohesin complexes might act in a coordinate manner with *KMT2D* and *TET2* to control enhancer functions.

Our data point to the lymphomagenic effect of *Smc3* deficiency manifesting specifically during late stages of GC exit when B-cells undergo terminal stages of plasma cell commitment. In general, differentiation requires that cells undergo various rounds of cell division. As cells exit from mitosis, cohesin is recruited to chromatin and regenerates the architectural features optimal for cell context dependent transcriptional programs to be maintained (63). Presumably post-mitotic architectural reconfiguration of the genome provides an opportunity to favor new architectural settings required for differentiation. Along these lines it is notable that we traced the effect of Smc3 haploinsufficiency to crucial, late cell divisions that give rise either to CD138^+^ plasma cells or CD138^–^ B-cells. This is consistent with a previous report showing that early events during PC differentiation, such as induction of *Irf4*, remain intact in *Smc3* ^wt/–^ B-cells, but late events such as *Prdm1* upregulation are impaired (6). We speculate that this leads to accumulation of greater numbers of mutated post GC B-cells, which may serve as the cell of origin of lymphomas observed in these mice. Hence it is possible that our findings could reflect loss of asymmetric division in B-cells as a potential mechanism of malignant transformation.

Finally, our results suggest that cohesin dose reduction contributes to lymphoma phenotypes in humans, in spite of the fact that cohesin mutations are uncommon in DLBCL. This is supported by the fact that DLBCLs enriched for lower expression of genes downregulated by *Smc3* haploinsufficiency also features reduced expression of cohesin core subunits as well as inferior clinical outcomes, an effect that was reproducible across two, large independent cohorts of patients. Moreover, and consistent with a previous report indicating that *Smc3* expression is a negative prognostic factor in DLBCL (6), we showed that reduction in the four core subunits *SMC3*, *STAG2*, *SMC1A* and *RAD21* are all independent adverse risk factors. What remains to be determined is the mechanism through which cohesin expression is suppressed in these tumors, as well as the reason why this may be the preferred rout to cohesin impairment instead of somatic mutations. Regardless, our data strongly support the notion that cohesin complex does play critical roles in lymphomagenesis and warrants further in-depth mechanistic study and consideration of potential therapeutic vulnerabilities.

## Data Availability Statement

The datasets presented in this study can be found in online repositories. The names of the repository/repositories and accession number(s) can be found below: https://www.ncbi.nlm.nih.gov/geo/, GSE172332.

## Ethics Statement

Ethical review and approval was not required for the study on human participants in accordance with the local legislation and institutional requirements. Written informed consent for participation was not required for this study in accordance with the national legislation and the institutional requirements. The animal study was reviewed and approved by Weill Cornell Medicine.

## Author Contributions

MR conceptualized, designed and performed research, analyzed and interpreted data, drafted the manuscript and supervised the study. CD analyzed RNA-sequencing, and single cell RNA-sequencing with the help of CC. AKl analyzed Hi-C. ZC analyzed patient data. BB analyzed exome capture. AKo analyzed whole genome sequencing. AV provided the Smc3 mouse model. CS and JB performed the RNA-sequencing of LPS-induced plasma cells. OE and CM provided expertise and resources for data analysis. AM participated in the study conceptualization, interpretation of data, drafting of the manuscript, acquisition of funds and supervision of the project. All authors contributed to the article and approved the submitted version.

## Funding

MR was a recipient of a postdoctoral fellowship grant from the Lymphoma Research Foundation and is funded by an NHLBI 5 T32 HL135465-3 training grant. AM is supported by NCI/NIH R35 CA220499, NCI/NIH P01 CA229086-01A1, LLS-SCOR 7012-16, LLS-TRP 6572-19, the Samuel Waxman Cancer Research Foundation, the Follicular Lymphoma Consortium and the Chemotherapy Foundation. AV is supported by NCI K08 CA215317, the William Raveis Charitable Fund Fellowship of the Damon Runyon Cancer Research Foundation (DRG 117-15) and an Evans MDS Young Investigator grant from the Edward P. Evans Foundation. CM thanks the Scientific Computing Unit, XSEDE Supercomputing Resources as well as the Starr Cancer Consortium (I7-A765, I9-A9- 071 and I13-0052) and acknowledges funding from the WorldQuant Foundation, The Pershing Square Sohn Cancer Research Alliance, the NIH (grants R01 CA249054, R01 AI151059, P01 CA214274, R01 AI125416-03 and R21 AI129851-02) and Leukemia and Lymphoma Society grants LLS 9238-16 and LLS MCL-982. AKo is supported by DP2GM123495 from the National Institutes of Health.

## Conflict of Interest

MR is a scientific advisor of NuRevelation, Inc. OE is supported by Janssen, Johnson and Johnson, Volastra Therapeutics, AstraZeneca and Eli Lilly research grants. He is scientific advisor and equity holder in Freenome, Owkin, Volastra Therapeutics and One Three Biotech. CM is a cofounder and board member for Biotia and Onegevity Health as well as an advisor or compensated speaker for Abbvie, Acuamark Diagnostics, ArcBio, Bio-Rad, DNA Genotek, Genialis, Genpro, Karius, Illumina, New England BioLabs, Qiagen, Whole Biome and Zymo Research. AM receives research funding from Janssen Pharmaceuticals, Sanofi and Daiichi Sankyo, has consulted for Epizyme and Constellation and is on the scientific advisory board of KDAC pharmaceuticals.

The remaining authors declare that the research was conducted in the absence of any commercial or financial relationships that could be construed as a potential conflict of interest. 

## Publisher’s Note

All claims expressed in this article are solely those of the authors and do not necessarily represent those of their affiliated organizations, or those of the publisher, the editors and the reviewers. Any product that may be evaluated in this article, or claim that may be made by its manufacturer, is not guaranteed or endorsed by the publisher.

## Supplementary Material

Supplementary Figure 1 Aberrant gene expression program in Smc3 haploinsufficient tumors. **(A)** RT-qPCR for Smc3 mRNA in Bcl6 (n=3) and Smc3/Bcl6 (n=3) tumors, normalized to Hprt1 mRNA expression. **(B)** Principal component analysis for Bcl6 and Smc3/Bcl6 tumor cell RNA-sequencing. **(C)** Dendrogram of unsupervised hierarchical clustering for Bcl6 and Smc3/Bcl6 tumor cell RNA-sequencing. **(D)** Normalized counts for cohesin subunits, cohesin regulators and Ctcf in Bcl6 (n=3) and Smc3/Bcl6 (n=6) tumors.

Supplementary Figure 2 Cohesin haploinsufficiency induces loss of interactivity of promoter-enhancers at tumor suppressor genes. **(A)** Contact maps at 100kb resolution for Bcl6 and Smc3/Bcl6 tumors depicting chromosomal interactions for the whole chromosome 14 (top panels) and for nucleotides 92 to 107 Mb in chromosome 14. **(B)** A (red) and B (blue) compartments genome wide (top panel) and for chromosome 14 in Bcl6 and Smc3/Bcl6 tumors (bottom panel), each at 100kb resolution. **(C)** Differential loop analysis ranked by log_2_ fold change showing either all significantly called loops genome wide (violet line) or all promoter-enhancer loops (black line). A normal distribution with mean equals 0 and standard deviation equals the standard deviation of all promoter-enhancer loops is depicted as green line. Top up and down regulated loops in Smc3/Bcl6 tumors are highlighted for promoter-enhancer loops. **(D)** Aggregate peak analysis (APA) of Hi-C-identified loops from Bcl6 (left) and Smc3/Bcl6 (right) tumor cells. The heatmaps were generated by using the raw chromatin interaction frequency**. (E)** Virtual 4C analysis showing normalized interactions with the *Kmt2d* or **(F)** *Dusp4* promoter for Bcl6 tumors (blue line) and Smc3/Bcl6 tumors (red line) at 20kb resolution. Loop calling significance following the Mango approach are shown for Bcl6 and Smc3/Bcl6 tumors with –log_10_(FDR). Enhancers were defined as H3K27Ac peaks mapped in germinal center B cells by Mint-ChIP. Rad21 ChIP-seq was performed in the mouse lymphoma cell line CH12.LX. The differences between normalized interactions with the *Kmt2d* **(E)** or *Dusp4* **(F)** promoter are shown as log_2_ fold-change between Bcl6 and Smc3/Bcl6 tumors. **(G)** RPKM values for *Ints12* gene in Bcl6 and Smc3/Bcl6 tumors. NS, non-significant differences.

Supplementary Figure 3 Smc3 haploinsufficient cells undergo proliferation burst but fail to differentiate into plasma cells. UMAP depicting cell lineage from CB → PC.

Supplementary Figure 4 Smc3 haploinsufficiency increases DNA damage in germinal center B cells. **(A)** Gating strategy used to detect phospho-γH2AX by flow cytometry in B cells, germinal centers, centroblasts, and centrocytes. **(B)** Freshly isolated resting B cells (control) and 90 Gy irradiated resting B cells (irradiated) were stained with anti- γH2AX antibodies and analyzed by FACS. Averages of percent positivity for γH2AX control and irradiated are shown in the plot below. **(C)** Scheme depicting the induced GC B cell culture system. **(D)** Representative karyotype arrangement produced from iGC B cells.

Supplementary Figure 5 Copy number and replication fork usage analysis in primary *Smc3* haploinsufficient germinal center B cells. **(A)** DNA copy number for mouse chromosomes 1 to 19 and ChrX in *Smc3* ^wt/wt^ (upper plot), *Smc3* ^wt/–^ (middle plot), and the difference (lower plot). **(B)** DNA replication timing for Chr 2 calculated by assessing the DNA copy number along the whole chromosome for *Smc3* ^wt/wt^ (blue line) and *Smc3* ^wt/–^ (green line).

Supplementary Figure 6 Decreased cohesin levels predict poor survival in DLBCL patients. **(A)** Dendrogram showing assignment of patients from the BCCA cohort to clusters 1 and 2, defined by unsupervised hierarchical clustering using the *Smc3* haploinsufficient gene signature. **(B)** Expression levels of cohesin core subunits in cluster 1 and cluster 2 in the BCCA cohort. **(C)** Kaplan-Meier overall survival curves for ABC-subtype DLBCL patients (n=108) in BCCA cohort clustered with the *Smc3* haploinsufficient signature (6). **(D)** Kaplan-Meier progression-free survival curves for ABC-subtype DLBCL patients (n=108) in BCCA cohort clustered with the *Smc3* haploinsufficient signature (6). **(E)** Dendrogram showing assignment of patients from the NCI cohort to clusters 1 and 2, defined by unsupervised hierarchical clustering using the *Smc3* haploinsufficient gene signature. **(F)** Expression levels of cohesin core subunits in cluster 1 and cluster 2 in the NCI cohort. **(G)** Kaplan-Meier overall survival curves for DLBCL patients (n=243) in NCI cohort clustered with the *Smc3* haploinsufficient gene signature (6).
