# Supplementary figures and images for: Cohesin Core Complex Gene Dosage Contributes to Germinal Center Derived Lymphoma Phenotypes and Outcomes
Source: Front Immunol. 2021 Sep 21;12:688493. doi: 10.3389/fimmu.2021.688493 (PMC8490713; doi:10.3389/fimmu.2021.688493)

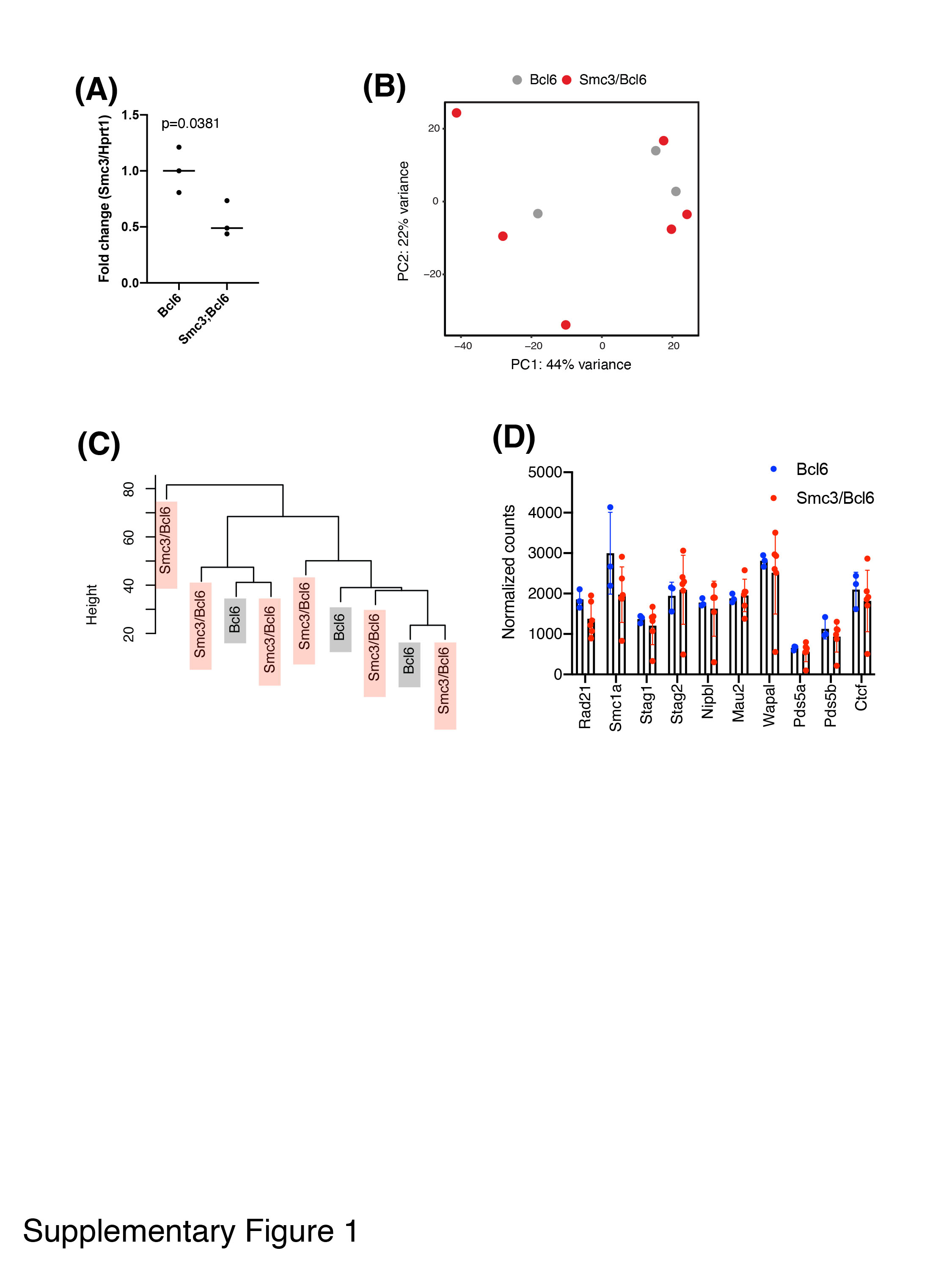

Supplement: Supplementary Figure 1 — Aberrant gene expression program in Smc3 haploinsufficient tumors. (A) RT-qPCR for Smc3 mRNA in Bcl6 (n=3) and Smc3/Bcl6 (n=3) tumors, normalized to Hprt1 mRNA expression. (B) Principal component analysis for Bcl6 and Smc3/Bcl6 tumor cell RNA-sequencing. (C) Dendrogram of unsupervised hierarchical clustering for Bcl6 and Smc3/Bcl6 tumor cell RNA-sequencing. (D) Normalized counts for cohesin subunits, cohesin regulators and Ctcf in Bcl6 (n=3) and Smc3/Bcl6 (n=6) tumors. [file Image_1.jpeg]

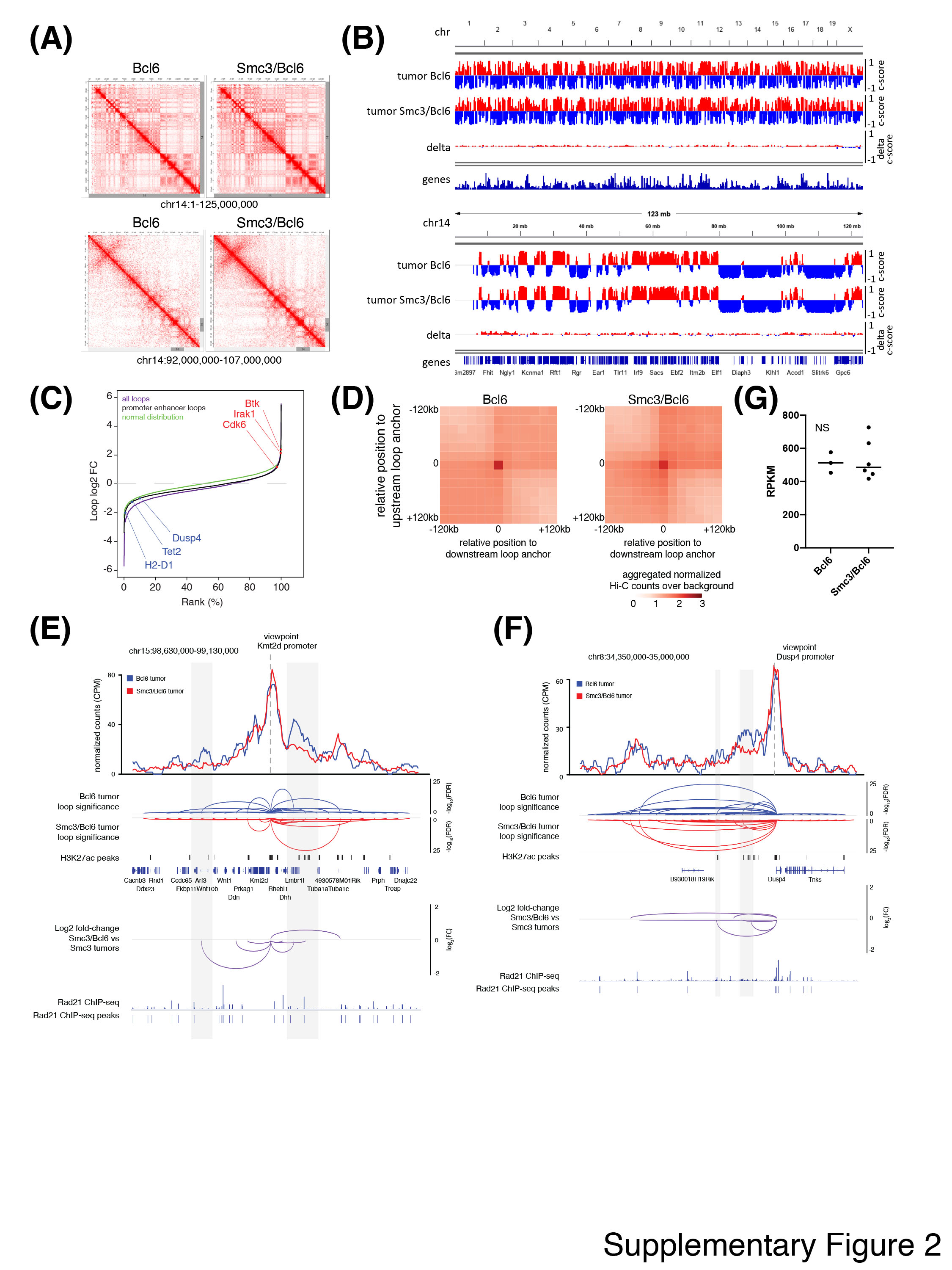

Supplement: Supplementary Figure 2 — Cohesin haploinsufficiency induces loss of interactivity of promoter-enhancers at tumor suppressor genes. (A) Contact maps at 100kb resolution for Bcl6 and Smc3/Bcl6 tumors depicting chromosomal interactions for the whole chromosome 14 (top panels) and for nucleotides 92 to 107 Mb in chromosome 14. (B) A (red) and B (blue) compartments genome wide (top panel) and for chromosome 14 in Bcl6 and Smc3/Bcl6 tumors (bottom panel), each at 100kb resolution. (C) Differential loop analysis ranked by log2 fold change showing either all significantly called loops genome wide (violet line) or all promoter-enhancer loops (black line). A normal distribution with mean equals 0 and standard deviation equals the standard deviation of all promoter-enhancer loops is depicted as green line. Top up and down regulated loops in Smc3/Bcl6 tumors are highlighted for promoter-enhancer loops. (D) Aggregate peak analysis (APA) of Hi-C-identified loops from Bcl6 (left) and Smc3/Bcl6 (right) tumor cells. The heatmaps were generated by using the raw chromatin interaction frequency. (E) Virtual 4C analysis showing normalized interactions with the Kmt2d or (F) Dusp4 promoter for Bcl6 tumors (blue line) and Smc3/Bcl6 tumors (red line) at 20kb resolution. Loop calling significance following the Mango approach are shown for Bcl6 and Smc3/Bcl6 tumors with –log10(FDR). Enhancers were defined as H3K27Ac peaks mapped in germinal center B cells by Mint-ChIP. Rad21 ChIP-seq was performed in the mouse lymphoma cell line CH12.LX. The differences between normalized interactions with the Kmt2d (E) or Dusp4 (F) promoter are shown as log2 fold-change between Bcl6 and Smc3/Bcl6 tumors. (G) RPKM values for Ints12 gene in Bcl6 and Smc3/Bcl6 tumors. NS, non-significant differences. [file Image_2.jpeg]

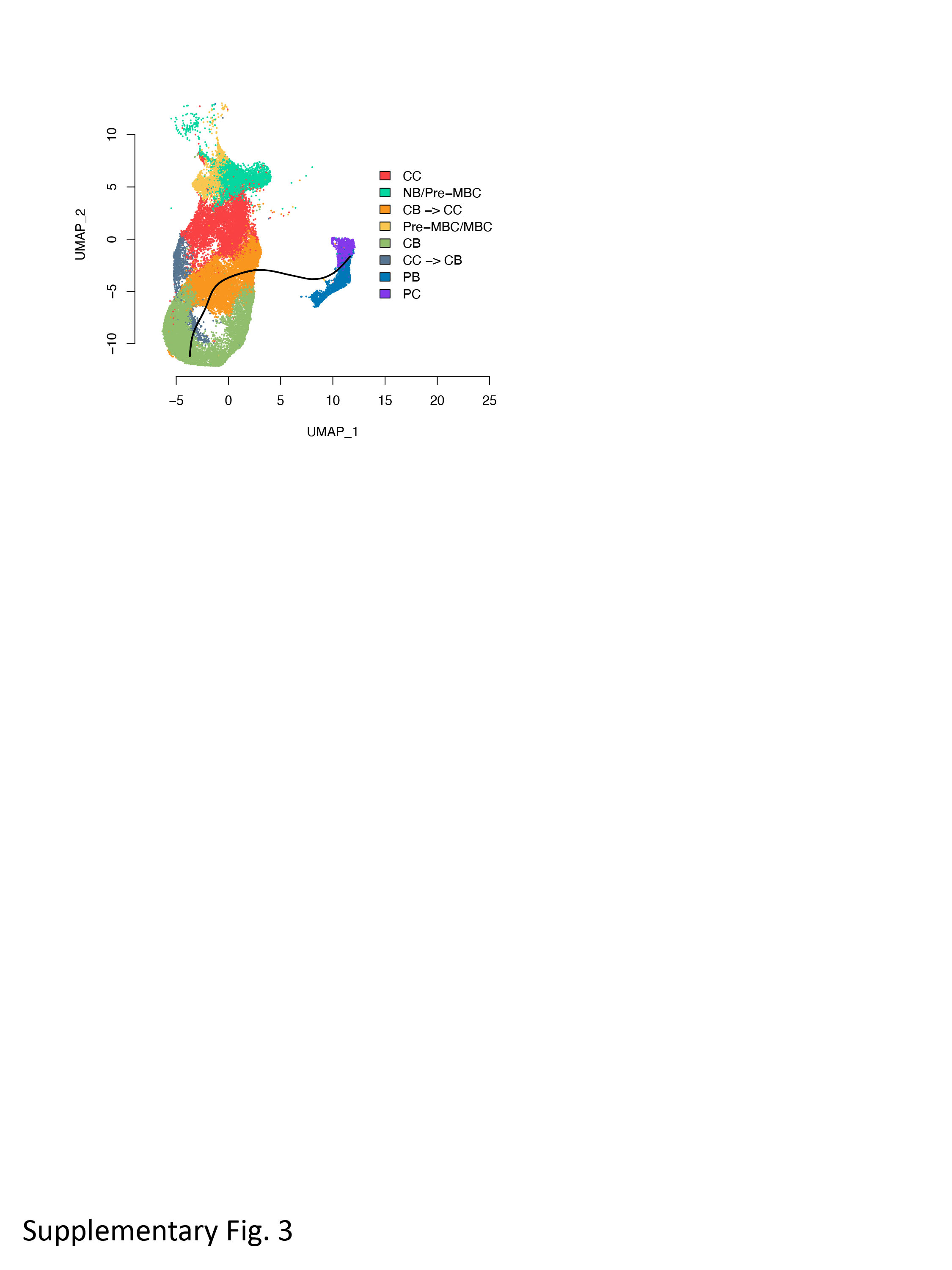

Supplement: Supplementary Figure 3 — Smc3 haploinsufficient cells undergo proliferation burst but fail to differentiate into plasma cells. UMAP depicting cell lineage from CB → PC. [file Image_3.jpeg]

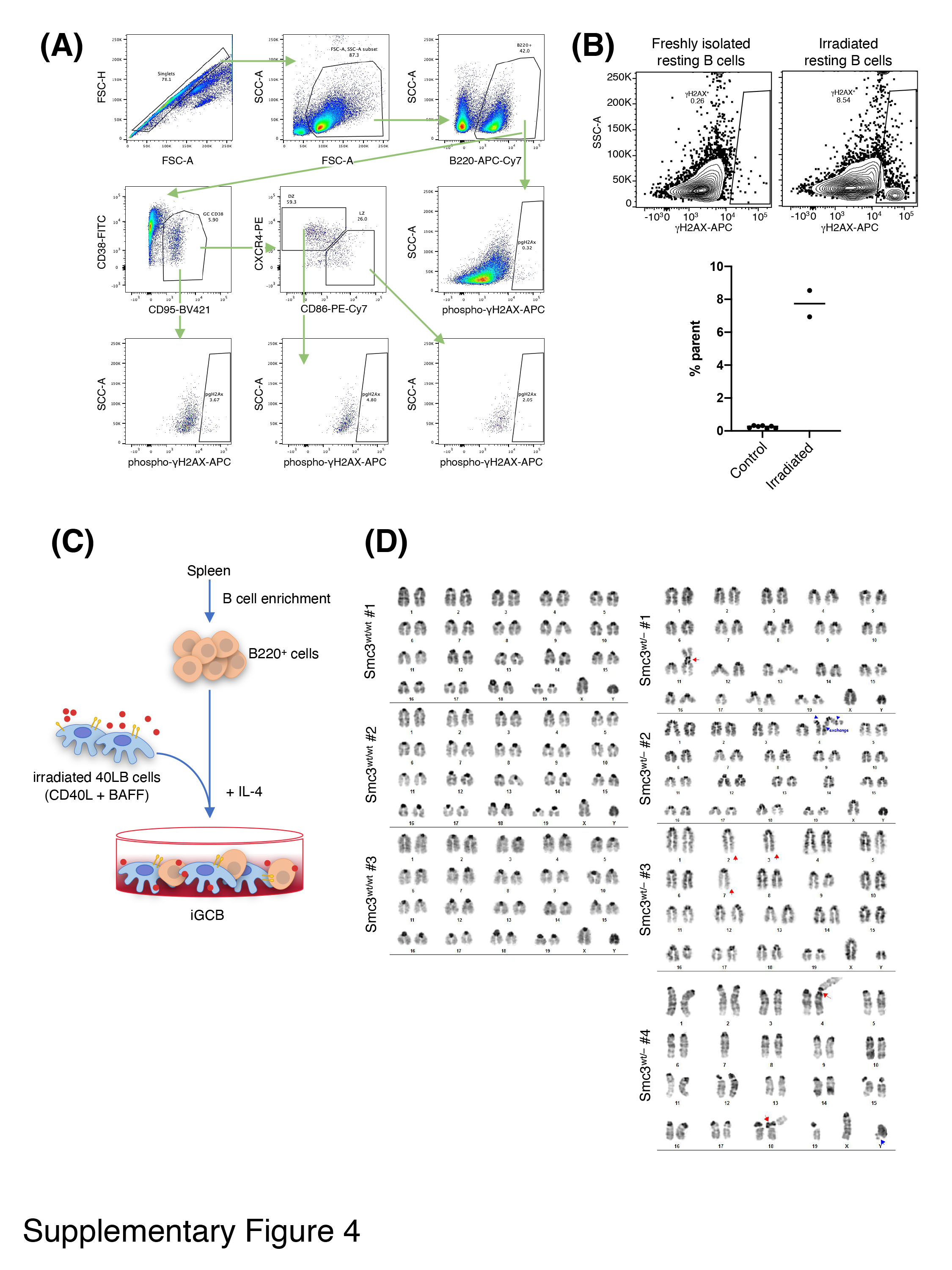

Supplement: Supplementary Figure 4 — Smc3 haploinsufficiency increases DNA damage in germinal center B cells. (A) Gating strategy used to detect phospho-γH2AX by flow cytometry in B cells, germinal centers, centroblasts, and centrocytes. (B) Freshly isolated resting B cells (control) and 90 Gy irradiated resting B cells (irradiated) were stained with anti- γH2AX antibodies and analyzed by FACS. Averages of percent positivity for γH2AX control and irradiated are shown in the plot below. (C) Scheme depicting the induced GC B cell culture system. (D) Representative karyotype arrangement produced from iGC B cells. [file Image_4.jpeg]

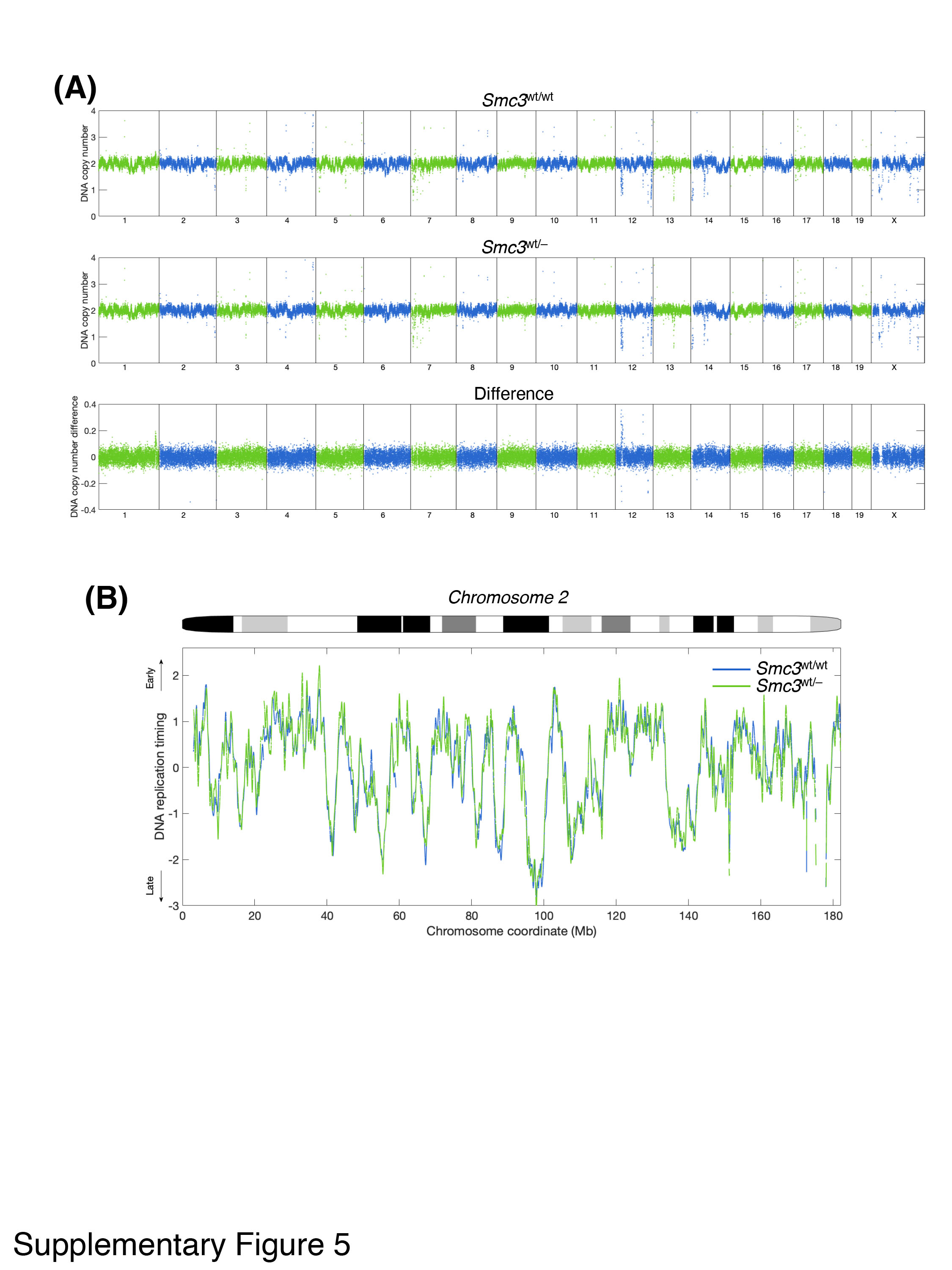

Supplement: Supplementary Figure 5 — Copy number and replication fork usage analysis in primary Smc3 haploinsufficient germinal center B cells. (A) DNA copy number for mouse chromosomes 1 to 19 and ChrX in Smc3 wt/wt (upper plot), Smc3 wt/– (middle plot), and the difference (lower plot). (B) DNA replication timing for Chr 2 calculated by assessing the DNA copy number along the whole chromosome for Smc3 wt/wt (blue line) and Smc3 wt/– (green line). [file Image_5.jpeg]

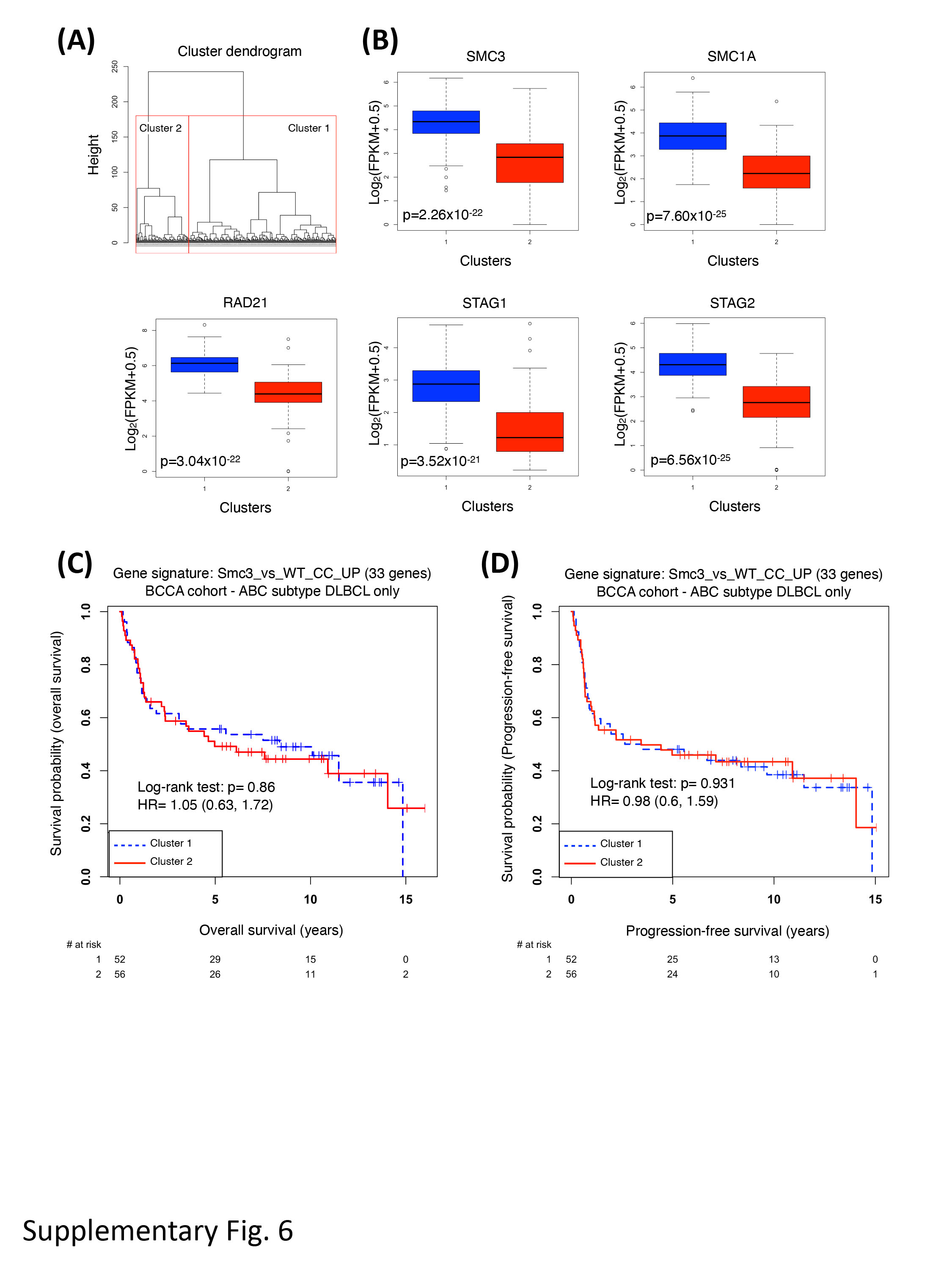

Supplement: Supplementary Figure 6 — Decreased cohesin levels predict poor survival in DLBCL patients. (A) Dendrogram showing assignment of patients from the BCCA cohort to clusters 1 and 2, defined by unsupervised hierarchical clustering using the Smc3 haploinsufficient gene signature. (B) Expression levels of cohesin core subunits in cluster 1 and cluster 2 in the BCCA cohort. (C) Kaplan-Meier overall survival curves for ABC-subtype DLBCL patients (n=108) in BCCA cohort clustered with the Smc3 haploinsufficient signature (6). (D) Kaplan-Meier progression-free survival curves for ABC-subtype DLBCL patients (n=108) in BCCA cohort clustered with the Smc3 haploinsufficient signature (6). (E) Dendrogram showing assignment of patients from the NCI cohort to clusters 1 and 2, defined by unsupervised hierarchical clustering using the Smc3 haploinsufficient gene signature. (F) Expression levels of cohesin core subunits in cluster 1 and cluster 2 in the NCI cohort. (G) Kaplan-Meier overall survival curves for DLBCL patients (n=243) in NCI cohort clustered with the Smc3 haploinsufficient gene signature (6). [file Image_6.jpeg]

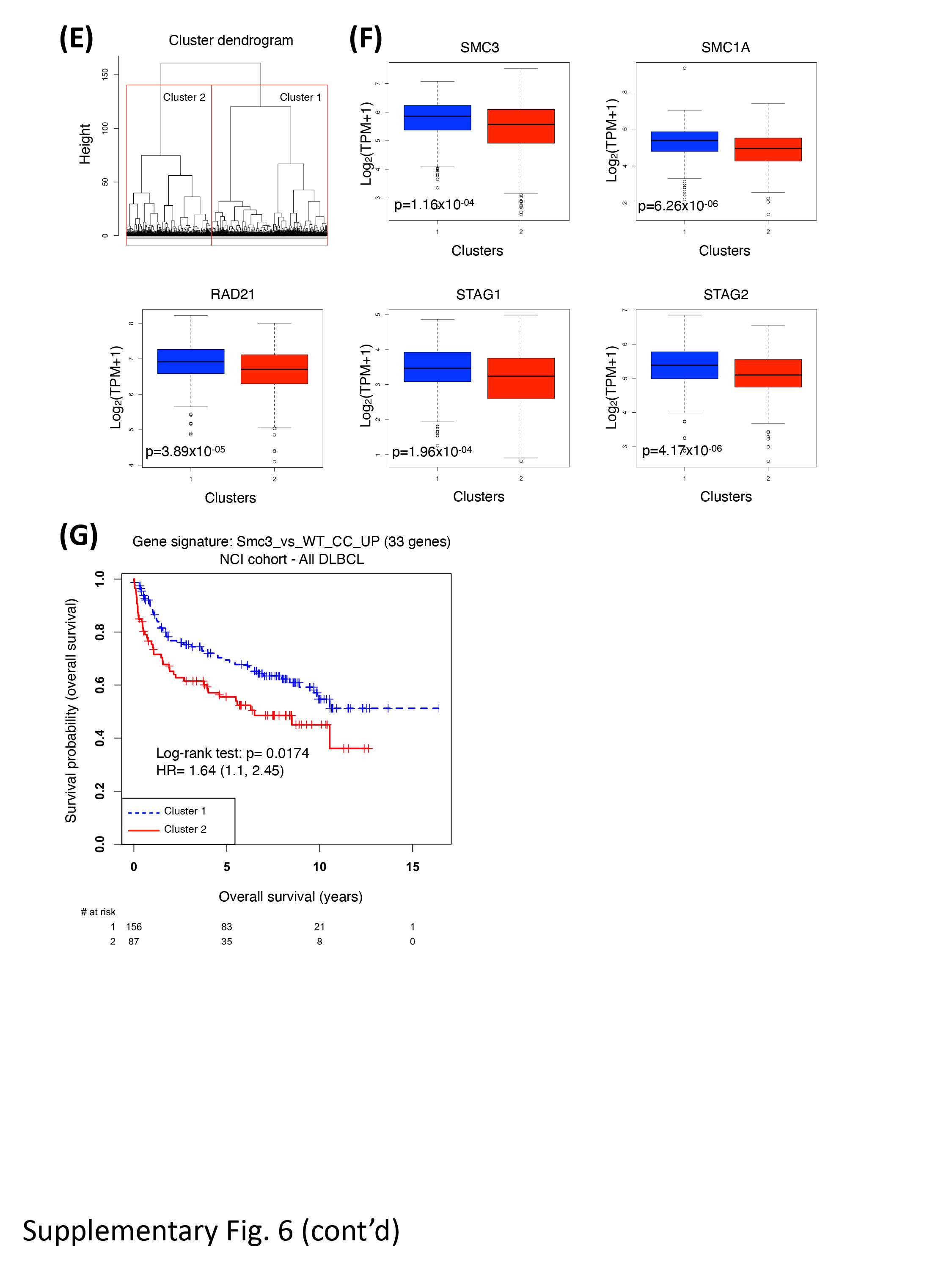

Supplement: Supplementary file 7 [file Image_7.jpeg]
